# TLR4 and CD14 trafficking and its influence on LPS-induced pro-inflammatory signaling

**DOI:** 10.1007/s00018-020-03656-y

**Published:** 2020-10-15

**Authors:** Anna Ciesielska, Marta Matyjek, Katarzyna Kwiatkowska

**Affiliations:** Laboratory of Molecular Membrane Biology, Nencki Institute of Experimental Biology of Polish Academy of Sciences, 3 Pasteur St., 02-093 Warsaw, Poland

**Keywords:** CD14, Endocytosis, Endotoxin, Endosome, LPS, TLR4

## Abstract

Toll-like receptor (TLR) 4 belongs to the TLR family of receptors inducing pro-inflammatory responses to invading pathogens. TLR4 is activated by lipopolysaccharide (LPS, endotoxin) of Gram-negative bacteria and sequentially triggers two signaling cascades: the first one involving TIRAP and MyD88 adaptor proteins is induced in the plasma membrane, whereas the second engaging adaptor proteins TRAM and TRIF begins in early endosomes after endocytosis of the receptor. The LPS-induced internalization of TLR4 and hence also the activation of the TRIF-dependent pathway is governed by a GPI-anchored protein, CD14. The endocytosis of TLR4 terminates the MyD88-dependent signaling, while the following endosome maturation and lysosomal degradation of TLR4 determine the duration and magnitude of the TRIF-dependent one. Alternatively, TLR4 may return to the plasma membrane, which process is still poorly understood. Therefore, the course of the LPS-induced pro-inflammatory responses depends strictly on the rates of TLR4 endocytosis and trafficking through the endo-lysosomal compartment. Notably, prolonged activation of TLR4 is linked with several hereditary human diseases, neurodegeneration and also with autoimmune diseases and cancer. Recent studies have provided ample data on the role of diverse proteins regulating the functions of early, late, and recycling endosomes in the TLR4-induced inflammation caused by LPS or phagocytosis of *E. coli.* In this review, we focus on the mechanisms of the internalization and intracellular trafficking of TLR4 and CD14, and also of LPS, in immune cells and discuss how dysregulation of the endo-lysosomal compartment contributes to the development of diverse human diseases.

## Introduction

The mammalian family of Toll-like receptors (TLR) consists of thirteen members with TLR4 being the most extensively studied one. TLRs are representatives of pattern recognition receptors (PRR), so named for their ability to recognize evolutionarily conserved components of microorganisms, including bacteria, viruses, fungi and parasites, collectively called pathogen-associated molecular patterns (PAMPs). The recognition of a PAMP by a PRR triggers rapid inflammatory reactions essential for the innate immunity, as discussed in several previous exhaustive reviews [1–5]. TLR4 is activated by lipopolysaccharide (LPS, endotoxin), a major component of the outer membrane of Gram-negative bacteria. During infection, TLR4 responds to the LPS present in tissues and the bloodstream and triggers pro-inflammatory reactions facilitating eradication of the invading bacteria [6]. It has been indicated that TLR4 can also be activated by endogenous compounds called damage-associated molecular patterns (DAMPs), including high mobility group box protein 1 (HMGB1) and hyaluronic acid. These compounds are released during tissue injury and can activate TLR4 in non-infectious conditions to induce tissue repair [7, 8]. Altogether, apart from LPS and its derivatives, up to 30 naturally occurring agonists of TLR4 with various chemical structures have been postulated. However, only three of them, Ni^2+^, the plant secondary metabolite paclitaxel, and disulfide HMGB1 have been demonstrated to be direct activators of TLR4, while the others can act as chaperones for TLR4 or promoters of LPS internalization [7, 9, 10]. Nevertheless, the impact of endogenous DAMPs on the TLR4 activity broadens the spectrum of pathophysiological conditions involving the TLR4-induced pro-inflammatory responses far beyond infectious diseases.

**Fig. 1:**
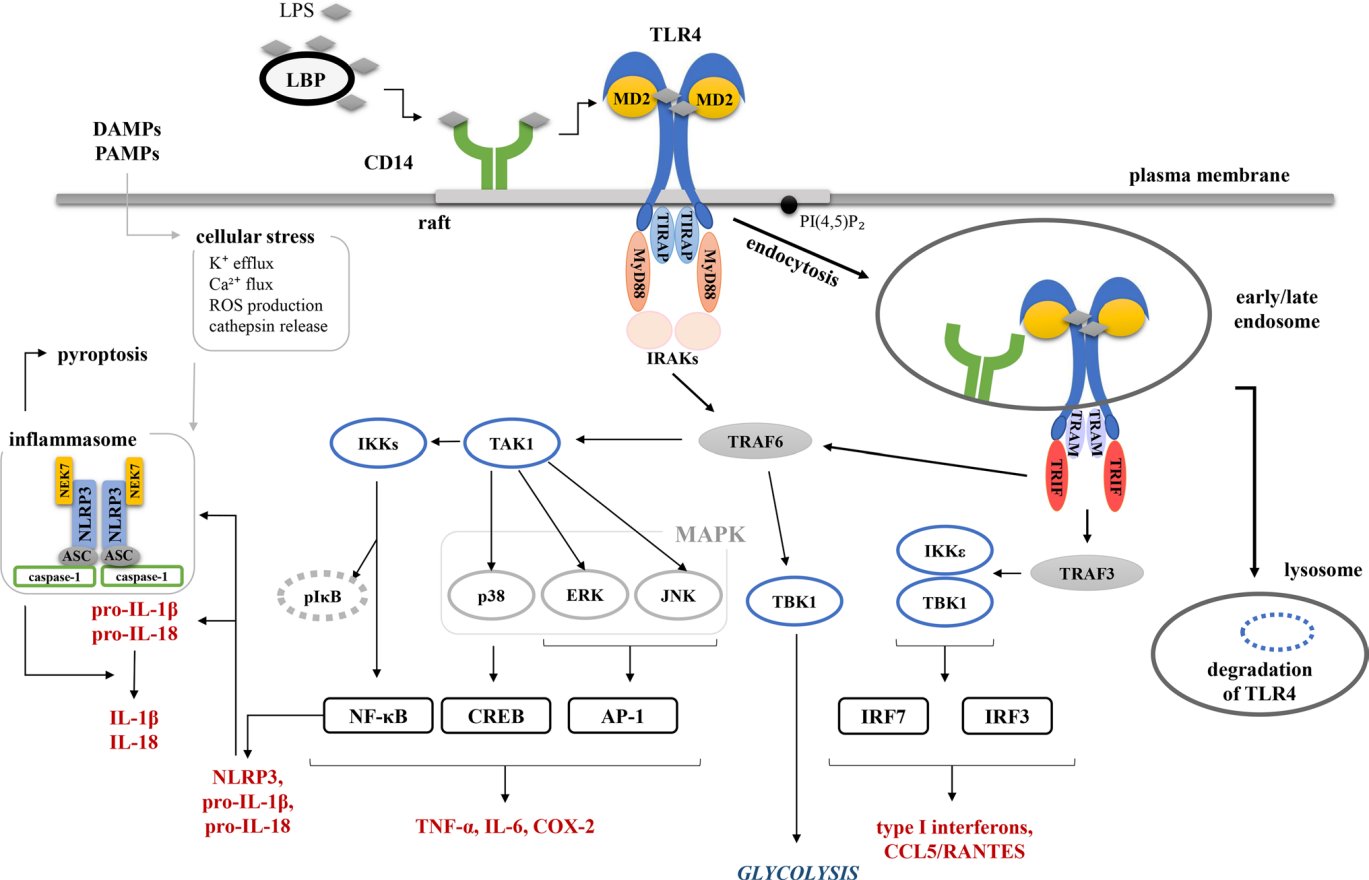
Pro-inflammatory signaling pathways of TLR4. TLR4 activates the MyD88-signaling pathway at the plasma membrane and after a CD14-dependent endocytosis initiates the TRIF-dependent cascade. Via activated NF-κB TLR4 also contributes to the activation of the cytosolic NLRP3 inflammasome. See text for details

TLR4 binds LPS with the help of LPS-binding protein (LBP) and CD14, and an indispensable contribution of the MD-2 protein stably associated with the extracellular fragment of the receptor (Fig. 1). The requirement for MD-2 for TLR4 activation by LPS was established shortly after the identification of TLR4 as the LPS receptor, since virtually no responses to LPS were detected in macrophages derived from MD-2^−/−^ mice [11, 12]. The binding of an LPS molecule to the TLR4/MD-2 complex involves acyl chains and phosphate groups of lipid A, the conserved part of LPS and the main inducer of pro-inflammatory responses to LPS [13, 14]. Hexa-acylated and diphosphorylated LPS, like *Escherichia coli* LPS (O111:B4), is one of the most potent agonists of TLR4 whereas under-acylated LPS and dephosphorylated LPS species have a weaker pro-inflammatory activity especially in human cells [15]. Structural determinants of this phenomenon are found in the TLR4/MD-2 complex and also in CD14 protein [13, 16], as discussed in the following sections.

Under-acylated and dephosphorylated LPS is synthesized by commensal bacteria which colonize human intestines, like *Bacteroides thetaiotaomicron*, which evade recognition by PRR [17] and are crucial for the maintenance of the intestinal immune balance [15]. In addition, the impermeability of the intestine epithelium to LPS is an important factor preventing its egress into the bloodstream [18]. On the other hand, during infection, deacylation and dephosphorylation of bacterial LPS is important for the termination of inflammatory responses, as discussed in detail below in the section concerning LPS detoxification.

An exaggerated and uncontrolled pro-inflammatory signaling triggered by TLR4 during infection can lead to sepsis, septic shock, and death [19]. Infections with Gram-negative bacteria, including *E. coli* and *Pseudomonas aeruginosa*, are the prevailing cause of severe sepsis in humans [20]. In addition, low doses of LPS derived from the gut microbiota can in certain conditions enter the bloodstream and evoke so-called metabolic endotoxemia leading to a chronic low-grade TLR4-dependent inflammation which contributes to the development of metabolic diseases, such as type 2 diabetes [18, 21–23]. Prolonged activation of TLR4 is also linked with several human hereditary and neurodegenerative diseases and also with autoimmune diseases and cancer [24, 25]. On the other hand, an experimental exclusion of TLR4-triggered signaling during low-grade polymicrobial sepsis resulted in an impaired bacterial clearance and thereby worsened organ injury leading to a higher mortality of mice [26, 27]. Thus, the cellular level of TLR4 and its signaling activity have to be tightly regulated to be beneficial rather than harmful to the host.

TLR4 is expressed in immune cells mainly of myeloid origin, including monocytes, macrophages and dendritic cells (DC), and also in some non-immune cells, like endothelial cells [28]. Most myeloid cells express also high amounts of plasma membrane-anchored CD14 [29, 30], which facilitates the activation of TLR4 by LPS and controls the subsequent internalization of the LPS-activated TLR4 important for receptor signaling and degradation. With the help of CD14, TLR4, unlike all the other TLRs, triggers two signaling pathways called the MyD88-dependent and the TRIF-dependent one after the adaptor proteins involved in their induction [31–34]. These signaling pathways lead to the production of two sets of pro-inflammatory cytokines which only partially overlap [35–39]. The MyD88- and TRIF-dependent signaling pathways are triggered consecutively and linked with the redistribution of the LPS-activated TLR4 from the plasma membrane to endosomes [40]. The translocation of TLR4 to the cell interior and its further lysosomal degradation facilitate termination of the inflammatory response [41].

In this review, we discuss the mechanisms of the internalization and intracellular trafficking of TLR4 and CD14, and also of LPS in immune cells. We emphasize the mechanisms regulating the trafficking of TLR4 and CD14 and thereby affecting the TLR4-induced pro-inflammatory signaling, its magnitude, duration and eventually also TLR4 degradation. Finally, we discuss data pointing to how disturbances in those processes contribute to the development of several human diseases.

## Signaling pathways triggered by TLR4

Activation of TLR4 by LPS is preceded by a chain of reactions which aim at converting LPS aggregates, derived from bacteria, into LPS monomers concentrated at the cell surface in the vicinity of the receptor (Fig. 1). These reactions are initiated by the serum LBP protein which binds to LPS aggregates (micelles) and, in the most typical scenario, facilitates subsequent extraction of LPS monomers by CD14 and the delivery of the LPS to the TLR4/MD-2 complex [42–44]. In agreement, blocking of LBP with an anti-LBP antibody inhibited the LPS-induced TLR4 signaling and endocytosis of the receptor [45]. Recently, the whole process of LPS transfer from micelles via LBP, CD14 to TLR/MD-2 was visualized at a single-molecule resolution [46]. That study indicated that one LBP molecule bound to an LPS micelle mediates several rounds of LPS transfer to CD14 molecules [46].

CD14 is a glycosylphosphatidylinositol (GPI)-anchored protein localized in nanodomains of the plasma membrane enriched in cholesterol and sphingolipids, so-called rafts, which are, therefore, considered as sites of TLR4 activation [3]. CD14 is detected predominantly on the surface of myeloid-lineage cells; however, low amounts are also found in non-myeloid cells, e.g., hepatocytes, adipocytes, corneal, and intestinal epithelial cells [29, 30, 47–51]. These latter cells produce mainly a soluble form of CD14 lacking the GPI anchor (sCD14) [47, 48]. However, mechanisms of the release of sCD14 were studied mainly in immune cells, where limited proteolysis of the membrane form of CD14 and also proteolysis-independent sCD14 formation have been detected [52]. The proteolysis of the membrane-bound CD14 can be carried out on the cell surface (so-called shedding) or intracellularly, after phagocytosis of bacteria when a following secretion of a truncated 13-kDa form of sCD14, called presepsin, has also been detected [53, 54]. The release of sCD14 that is independent of its membrane-bound form should not be neglected since patients suffering from paroxysmal nocturnal hemoglobinuria with defects in GPI anchor synthesis, who do not express membrane-anchored CD14, have normal levels of serum sCD14 [55, 56].

Both membrane-bound and soluble CD14 can transfer the LPS molecule to the TLR4/MD-2 complex [44, 46, 57]. LPS is bound in the N-terminal hydrophobic pocket of CD14 which differs in some details of structure between human and murine CD14 [16, 58]. The CD14 hydrophobic pocket probably accommodates up to five acyl chains of the endotoxin while the remaining one can facilitate the association of the CD14-LPS complex with MD-2 [16]. It has been found recently that the transfer of LPS from CD14 to MD-2 in the TLR4/MD-2 complex is facilitated by TLR4 which probably forms a transient intermediate with CD14, LPS or both [46].

A crystallographic analysis of the human TLR4/MD-2 complex with LPS bound has revealed that five of the six acyl chains of LPS are buried in the hydrophobic pocket of MD-2 while the sixth one interacts with TLR4 of another TLR4/MD-2 complex, and that the dimerization of the TLR4/MD-2 complexes is strengthened by ionic bonds between the phosphate group of lipid A and the neighboring TLR4 molecule [13]. Accordingly, removing of one or two acyl chains from the LPS molecule converts it from an agonist to an antagonist of human TLR4, as was shown using LPS of *Neisseria meningitidis* H44/76*, Pseudomonas aeruginosa,* and *Yersinia pestis* [59–61]. Interestingly, murine TLR4 is activated with similar efficiency by tetra-, penta- and hexa-acylated LPS from the same bacteria [59–61]. A contrasting ability to activate human and murine TLR4/MD2 complex has also been shown for tetra-acylated lipid IVa which turned out to be an antagonist for the former and a weak agonist for the latter [62]. The agonistic activity of lipid IVa toward murine TLR4 is due to its unique ionic interactions at the dimerization interface of the murine receptor that cannot be formed with human TLR4 [63]. Moreover, the length of the acyl chains as well as their saturation also seem important for the pro-inflammatory activity of LPS, as has been postulated based on studies of the immunomodulatory activity of lipid A and cardiolipin analogues [64, 65]. Accordingly, penta-acylated diphosphorylated LPS with one unsaturated acyl chain from *Rhodobacter spheroides* and its synthetic tetra-acylated lipid A derivative, Eritoran, are antagonists for both human and mouse TLR4 [66–68].

The interaction of TLR4/MD-2 with two molecules of an agonistic LPS species induces dimerization of the ectodomains of two TLR4 molecules which acquire an “M-shape” with their intracellular fragments put in juxtaposition [12, 13, 69]. Each intracellular fragment contains a Toll/IL-1R homology (TIR) domain prone to homotypic interactions with TIR domains of four adaptor proteins. When in the plasma membrane, TLR4 interacts with the first adaptor pair, TIRAP (also called Mal [70]) and MyD88 [71, 72]. Apart from the TIR domain, TIRAP also carries a domain enriched in basic and aromatic residues that interacts with phosphatidylinositols (PIs) and phosphatidylserine (PS) [72–74]. In the plasma membrane, TIRAP can bind both PS and phosphatidylinositol 4,5-bisphosphate (PI(4,5)P_2_) but only the latter interaction is required for TLR4 signaling [72, 73]. The TLR4-bound TIRAP recruits MyD88 which further binds interleukin-1 receptor-associated kinase (IRAK) 1 and 2 and a submembrane signaling complex called the myddosome is formed [72, 75, 76]. The assembled myddosome recruits E3 ubiquitin ligase TRAF6, which triggers a signaling cascade involving TAK1 kinase and leading, through the phosphorylation and activation of IκB kinases α/β (IKKα/β), to nuclear translocation of the NF-κB transcription factor. In addition, downstream of TRAF6 and TAK1, MAP kinases are phosphorylated to activate transcription factors AP-1 and CREB [77]. The signaling complex of TLR4 containing TIRAP and MyD88 also activates type I PI3-kinase which phosphorylates PI(4,5)P_2_ to phosphatidylinositol 3,4,5-trisphosphate (PI(3,4,5)P_3_) and triggers activation of Akt [74, 78]. Collectively, the MyD88-dependent signaling pathway induces expression of genes encoding pro-inflammatory mediators, such as tumor necrosis factor α (TNF-α), interleukin (IL)-6, cyclooxygenase 2, and type III interferons (IFNλ1/2), the latter contributing to epithelial barrier integrity, which is crucial for host defense [35, 79–81]. In addition, the MyD88-dependent signaling pathway also participates in the production of anti-inflammatory mediators, like IL-10 helping to terminate the inflammation [35, 82]. It is worth mentioning that several factors negatively affect the MyD88-depenedent signaling cascade to prevent an exaggerated production of pro-inflammatory mediators. These include A20 ubiquitin-modifying enzyme, the BCAP-PI3-kinase–Akt axis, and Lyn tyrosine kinase [83–86].

Recent studies indicate that the LPS-induced MyD88-dependent signaling triggers and also modulates the cell metabolism. It was shown that TRAF6 interacts with TBK1, the kinase which in turn activates Akt kinase leading to a rapid enhancement of glycolysis [87, 88]. The LPS-stimulated glycolysis and subsequent synthesis of acetyl-CoA and de novo synthesis of fatty acids can facilitate, respectively, histone acetylation required for gene transcription and expansion of the endoplasmic reticulum and Golgi apparatus necessary for intense production and secretion of cytokines [87, 89].

The MyD88-dependent signaling is followed by internalization of TLR4. Simultaneously, TIRAP and MyD88 dissociate from the membrane allowing TLR4 to bind in the endosome a second set of TIR-containing adaptor proteins, TRAM and TRIF [90]. In this tandem, TRAM is a bridging adaptor facilitating the interaction of TRIF with TLR4 [91]. In unstimulated cells, both TLR4 and TRAM localize to the plasma membrane, endosomes, endocytic recycling compartment (ERC), and the Golgi apparatus [40, 92–95]. However, the presence of TRAM and TLR4 in the same cellular compartment is not sufficient for their interaction. Detailed studies have revealed that in the plasma membrane, TRAM localizes to regions enriched in CD14 rather than TLR4 and stimulation of cells with LPS induces the interaction between TLR4 and TRAM in endosomes [94]. TRAM binds to membranes via a myristoyl residue attached to Gly2 (after the removal of the N-terminal methionine) and via a neighboring polybasic domain that mediates its interaction with various PIs and phosphatidic acid [40]. Both regions are required for the targeting of TRAM to the plasma membrane; however, only the *N*-myristoylation governs its location to endosomes [40]. Furthermore, only the *N-*myristoylation is indispensible for the TRAM involvement in the endosomal signaling pathway of TLR4 [40, 93]. Non-myristoylated Gly2Ala TRAM acted as a dominant negative mutant interfering with this signaling pathway of TLR4 [93].

The TRIF-dependent signaling pathway of TLR4 includes activation of the ubiquitin ligase TRAF3 followed by the activation of non-canonical IKK kinases: TANK binding kinase 1 (TBK1) and IKKε. TBK1 phosphorylates the pLxIS consensus motif of TRIF that is necessary to recruit interferon regulatory factor (IRF) 3. IRF3 is also phosphorylated by TBK1 and then dissociates from TRIF, dimerizes and translocates to the nucleus [96]. Finally, IRF3 and to a lower extent also TBK1-activated IRF7 induce expression of genes encoding type I IFN, the chemokine CCL5/RANTES and interferon-regulated genes as that encoding the chemokine CXCL10/IP-10 [35, 80, 91, 97]. This pathway also participates in the production of the anti-inflammatory interleukin IL-10 [98]. In addition, TRIF induces late activation of NF-κB via recruitment and activation of TRAF6 [99] or receptor-interacting serine/threonine-protein kinase 1 (RIPK1) [100]. RIPK1 interacts with the RIP-homotypic motif (RHIM) present in the C-terminus of TRIF, which is also recognized by RIPK3 [101]. RIPK1 and RIPK3 are engaged in LPS-induced ERK1/2 activation and cytokine production but in certain conditions can also mediate necrotic cell death [102–104]. Ultimately, TLR4 is degraded in lysosomes [41].

It has recently been established that TLR4 is also involved in so-called canonical activation of the NRLP3 inflammasome. The LPS-induced activation of this inflammasome via the canonical and also via a non-canonical, hence not involving TLR4, pathways has been intensively studied and discussed in excellent reviews [105–108]. In brief, the NLRP3 inflammasome is a multimeric complex of NRLP3, ASC, NEK7 and pro-caspase-1, which ultimately serves to promote autoproteolysis and activation of caspase-1. In turn, caspase-1 cleaves pro-IL-1β and pro-IL-18 to their secreted forms. Caspase-1 also catalyzes the proteolysis of gasdermin D which binds to the inner surface of the plasma membrane and forms pores which can lead to cell death via pyroptosis or release of IL-1β from living cells [109]. Activation of TLR4 in human and murine macrophages serves as a priming signal inducing expression of NRLP3 and pro-IL-1β and posttranslational modifications of NRLP3. The assembly of a fully active inflammasome is triggered by a second stimulus provided by diverse PAMPs and DAMPs. The exact mechanism leading to the inflammasome formation in response to these structurally different molecules is not known, but they all seem to induce cellular stress manifested by, e.g., K^+^ efflux, mitochondria dysfunction, lysosome damage, which is then somehow detected by NLRP3. Since the expression of NRLP3 is triggered in an NF-κB-dependent manner, both signaling pathways of TLR4 can in principle be involved. Accordingly, caspase-1 cleavage and activation occurred in LPS-stimulated macrophages derived from MyD88- or TRIF-knock-out mice while the double knock-out did not support it [110, 111].

Activation of the non-canonical inflammasome does not require TLR4 participation, but is induced by LPS present in the cytosol. LPS directly binds and activates caspase-11 (human caspase-4 and -5) which next activates the primed NLRP3 inflammasome and induces IL-1 release and pyroptosis. It is unclear how LPS enters the cytosol; however, recent studies indicate that it can escape from endosomes which are formed during internalization of LPS-rich outer membrane vesicles (OMV) derived from Gram-negative bacteria or from phagosomes enclosing the bacteria [112]. It can also be internalized by the receptor for advanced glycation end-products (RAGE) after binding to HMGB1 [113].

In addition, in human and porcine monocytes (but not in murine cells), LPS induces NLRP3 activation and IL-1β release without the second stimulus [114]. Gaidt et al. called this phenomenon the “alternative inflammasome” and found that it is induced by TLR4-dependent activation of the TRIF-RIPK1-capsase-8 axis upstream of NLRP3 and caspase-1. While it ultimately leads to IL-1β secretion, no concomitant pyroptosis of monocytes occurs [115]. Recently, the alternative activation of inflammasome by apolipoprotein C3 and TLR2/TLR4 heterodimer has been described, pointing to its involvement in sterile inflammation triggered by triglyceride-rich lipoproteins [116].

In addition to LPS-induced cytokine production, stimulation of macrophages and DC with the endotoxin induces of adaptive immune responses which are executed by T and B lymphocytes. The initiation of the adaptive immune responses follows activation of various TLRs and involves antigen presentation in the context of major histocompatibility complex (MHC) class I and II, as reviewed by [5, 117]. It has been established that activation of TLR4 by LPS in both murine DC and macrophages induces upregulation of costimulatory molecules (CD40, CD80, and CD86) on the cell surface which are required for antigen presentation for T lymphocytes. However, significant differences were found in the signaling pathways leading to this phenomenon. Namely, in macrophages, the upregulation depends strictly on the TRIF-dependent pathway, whereas in DC both the MyD88- and TRIF-dependent ones are involved [118–120]. The increased cell surface presence of the costimulatory molecules and also of MHC II is a hallmark of DC maturation required for antigen presentation by these cells. LPS upregulates the cell surface level of MHC II at various steps of the formation of antigen-MHC II complexes, including enhanced lysosomal acidification, proteolysis yielding antigens, and transport of the complexes to the surface of DC [121, 122]. Beside the contribution to the upregulation of MHC II and costimulatory molecules, the TLR4-triggered MyD88-dependent signaling in DC also induces production of cytokines leading to Th1 cell polarization and also facilitates fusion of MHC I-bearing recycling endosomes with phagosomes to allow cross-presentation of antigens during infection [123].

The signaling pathways triggered by TLR4 described above are best characterized in monocytes, macrophages, and DC; however, it has to be emphasized that certain aspects of the LPS-induced responses vary between DC and macrophages. In murine DC, LPS induces activation of nuclear factor of activated T cells (NFAT) and apoptosis of fully matured DC [124]. Also in these cells, CD11b contributes significantly to TLR4 activation [125]. Additionally, murine DC vary from human ones since only selected subtypes of the latter expresses TLR4 and CD14, as described in more detail in the next chapter.

A cell specificity of the LPS-induced responses is also observed in other immune cells, including human and murine granulocytes, mast cells, and lymphocytes which express low amounts of TLR4. Among those cells, CD14 is found in neutrophils and murine basophils [29, 30, 126–129]. Thus, due to a lack of CD14 and low content of TLR4/MD-2 and TRAM in mast cells, the TRIF-dependent pathway could not be detected in cells of murine origin [130]. Therefore, these cells stimulated with LPS produce TNF-α but not IFN-β [131, 132]. Activation of TLR4 in neutrophils leads to ROS generation, cytokine production and other responses which are cell specific, like autophagy and modulation of cell survival [30, 133–136]. In addition, in murine neutrophils, LPS induces production and secretion of histamine in a PI3K-dependent manner [137]. Moreover, LPS can stimulate neutrophils to form NETs (neutrophil extracellular traps), i.e., extracellular fibers composed of decondensed chromatin and granule proteins which trap bacteria allowing their extracellular killing [138–140].

In summary, TLR4 activated by LPS triggers two consecutive signaling pathways which are correlated with a redistribution of the receptor: the MyD88-dependent signaling is triggered by TLR4 localized to the plasma membrane, while the TRIF-dependent one by the TLR4 internalized in endosomes. The two pathways lead to a synchronized production of pro- and anti-inflammatory mediators, contribute to the activation of NLRP3 inflammasome, modulate cell metabolism, initiate adaptive immune responses and other cell type-specific reactions; eventually lysosomal degradation of TLR4 takes place. This course of events suggests that the TLR4-induced pro-inflammatory reactions can be regulated by the rate of its endocytosis and trafficking through the endo-lysosomal compartment.

## Mechanisms controlling internalization of TLR4

Endocytosis of TLR4 is required for the TRIF-dependent pro-inflammatory signaling to occur and also for the following degradation of the receptor and termination of the signaling [40, 41]. Somewhat surprising, it has been established that the LPS-induced internalization of TLR4 is independent of its signaling activity. Thus, a TLR4 mutant lacking the intracellular part, hence the TIR domain, underwent internalization despite being unable to trigger myddosome formation in murine bone marrow-derived macrophages (BMDM) stimulated with LPS. The amount of the truncated receptor in the plasma membrane progressively declined, similarly as in the case of wild type TLR4 [17]. On the contrary, the extracellular domain of TLR4 was indispensable for its LPS-induced endocytosis and further studies have indicated that in fact the interaction of MD-2 with CD14 drives the uptake of the receptor. Thus, the impact of MD-2 on LPS-induced signaling stems from its role in the dimerization of the TLR4/MD-2 complexes during LPS binding and the contribution to the endocytosis of TLR4 [17].

In most cases, the LPS-induced internalization of TLR4 is controlled by CD14 (Tab. 1) [32, 33]. Exceptions include TLR4 endocytosis followed by the TRIF-dependent signaling induced by a TLR4/MD-2 agonistic antibody (UT12) or a synthetic small-molecule TLR4 ligand (1Z105) [141]. And while phagocytosis of the Gram-negative *E. coli* did occur in DC derived from CD14-knock-out mice [33], no LPS-induced endocytosis of TLR4 took place in DC or BMDM derived from those animals. Accordingly, the TRIF-dependent signaling was abolished, while the MyD88-dependent one was not affected by the CD14 depletion especially in cells stimulated with so-called rough chemotype (devoid of the *O*-polysaccharide chain) of LPS [31, 32, 34]. In cells poor in CD14, such as murine splenic B lymphocytes, TLR4 does not undergo endocytosis [33]. The dependence of TLR4 internalization on CD14 has been confirmed by recent studies on CD14 glycosylation. Inhibition of CD14 core fucosylation caused by depletion of α-(1,6)-fucosyltransferase impaired CD14 and TLR4 endocytosis and thereby the TRIF-dependent signaling. It was found that the interference with CD14 fucosylation led to a reduction of its cell surface level which was considered the main reason of the impaired TLR4 endocytosis [142, 143]. Conversely, the increase of CD14 level during maturation of murine DC accelerates the LPS-induced endocytosis of TLR4 [33].

**Table 1:** Effects of down-regulation of selected proteins involved in TLR4 endocytosis and degradation on TLR4 internalization and signaling

Protein^a^	Effect on			Cells	References
	TLR4 internalization	MyD88-dependent pathway	TRIF-dependent pathway		
CD14	↓	↓	↓	macrophages, DC	[17, 33, 160]
TRAM	↓	nd	↓	HEK293, macrophages	[40, 93]
PLC-γ2/Ca^2+^	↓	↑	↓	macrophages	[33]
	↓	–	↓	DC	[33, 150]
Syk	↓	–	↓	macrophages	[33]
PI3K p110δ	↓	↑	↓	DC	[74]
Arf6	↓	↓	↓	J776, Raw264.7, macrophages, DC	[73, 152, 153]
Dynamin^b^	↓	–	↓	DC, macrophages, Raw264.7	[40, 74, 155]
	↓	↓	↓	J774, actrocytes	[159, 160]
Dynamin	nd	↑	nd	HEK293	[41]
Clathrin^b^	↓	↓	↓	Raw264.7, J774, macrophages	[155, 160, 265]
	↓	–	↓	astrocytes	[159]
Dab2	↑	–	↑	Raw264.7	[166]
Hrs	nd	↑	nd	HEK293, monocytes	[41]
Lyst^c^	–	–	↓	macrophages, DC	[183]
Vps33B	–	↑	↑	macrophages	[180]
GMFG^d^	↓	↑	↑	macrophages	[196]
Rab7a	nd	nd	↓	macrophages	[183]
Rab7b^d^	–	↑	↑	Raw264.7, macrophages	[184]
Rab11	nd	–	↓	monocytes, HEK293	[95]
Rab10	↓	↓	↓	macrophages, Raw264.7	[179]
TMED7	nd	–	↑	HEK293, THP-1, macrophages, PBMCs	[182]
TAG	nd	–	↑	HEK293, THP-1, PBMCs	[181]

**↓** Down-regulation, **↑** upregulation, *nd* no data

^a^In most cases, proteins were down-regulated by knock-out or knock-down of encoding genes

^b^Inhibition of the protein caused by a drug treatment

^c^Deleterious mutation

^d^Overexpression of the protein had opposite effects on the MyD88- and TRIF-dependent pathways

Notably, it has been established that CD14 undergos low-rate constitutive endocytosis also in unstimulated cells [17]. The decreasing pool of cell-surface CD14 is replenished by the newly synthesied protein. Binding of LPS to TLR4/MD-2 and the concomitant interaction of MD-2 with CD14 converts the TLR4/MD-2/LPS complex into a cargo of CD14. Simultanously, the rate of CD14 internalization is accelerated [17, 144]. On the basis of those data, CD14 has earned the name of “transporter associated with the execution of inflammation” (TAXI) [17].

The mechanism of the CD14-dependent internalization of TLR4 remains to be revealed, but it is likely linked with the raft localization of CD14, activation of the Syk tyrosine kinase, and local turnover of PI(4,5)P_2_ [33, 50, 145]. CD14 triggers biphasic generation of PI(4,5)P_2_ in LPS-stimulated macrophages and the newly generated PI(4,5)P_2_ accumulates in the raft fraction of these cells. The PI(4,5)P_2_ generation correlated with a biphasic activation of NF-κB and was required for maximal production of cytokines in the both signaling pathways of TLR4 [145, 146]. An increase of the PI(4,5)P_2_ level can be needed for the recruitment of TIRAP to the plasma membrane, while the following PI(4,5)P_2_ hydrolysis/phosphorylation is required for the TLR4 endocytosis to occur. The uptake of TLR4 can be initiated by the Syk kinase which binds to the immunoreceptor tyrosine-based activation motif of DAP12 which is an adaptor of CD300b receptor, and/or the γ chain of Fcε receptor [147]. Subsequently, Syk activates phospholipase Cγ2 (PLCγ2). Indeed, inhibition/knock-down of Syk or PLCγ2 abolished TLR4 internalization in LPS-stimulated murine BMDM and DC [33, 50, 147]. It deserves to be mentioned that Syk/PLC-γ2 inhibitors blocked not only the LPS-, but also the UT12-induced endocytosis of TLR4 that proceeded without a CD14 involvement [141]. PLCγ2 cleaves PI(4,5)P_2_ to DAG and inositol 1,4,5-trisphosphate (IP_3_) which induces Ca^2+^ release from the endoplasmic reticulum. Inhibition of this process by 2-ABP, an antagonist of IP_3_ receptor localized predominantly in the endoplasmic reticulum, blocked TLR4 endocytosis and the TRIF-dependent signaling [148]. PI(4,5)P_2_ is also a substrate for class I PI3-kinase which phosphorylates it to PI(3,4,5)P_3_. Mutation of the p110δ catalytic subunit of PI3-kinase in murine DC slowed down the rate of LPS-induced endocytosis of TLR4 while phagocytosis of *E. coli* was not affected [74]. In addition, CD14 controls the influx of extracellular Ca^2+^ mediated by TRPM7, a plasma membrane cation channel with kinase activity. A knock-out of TRPM7 inhibited endocytosis of CD14/TLR4 in murine BMDM. Furthermore, phosphorylation and nuclear translocation of IRF3 and also of NF-κB were inhibited and the following production of the TRIF-dependent cytokines was reduced. These processes were depended on the influx of extracellular Ca^2+^ rather than on the release of Ca^2+^ from intracellular stores, although an input of the latter to the regulation of TLR4-induced signaling was not precluded [149].

Since the downstream effectors of Syk, PLCγ2 and PI3-kinase, are involved in a broad spectrum of cellular processes [74, 150, 151], their activity is likely to affect various other aspects of TLR4-induced signaling in addition to the receptor endocytosis. Nevertheless, the impaired internalization of TLR4 observed after inhibition/mutation/knock-down of those enzymes correlated with a down-regulation of the TRIF-dependent signaling reflected by reduced IRF3 phosphorylation and impaired expression of genes encoding ISG54/IFIT-2, TRAIL, CCL5/RANTES, and IFN-β. The MyD88-dependent production of cytokines was not affected or was even elevated at the expense of the TRIF-dependent one [33, 74, 148].

Another important protein involved in PIs turnover taking part in the LPS-induced TLR4 endocytosis and signaling is the small GTPase ADP-ribosylation factor 6 (Arf6). Arf6 activates PI4-phosphate 5-kinase type Iα (PIP5KIα) which phosphorylates PI(4)P to PI(4,5)P_2_ [73]; therefore, its activity can modulate TLR4 signaling, endocytosis and trafficking. Indeed, PIP5KIα and PIP5KIγ were found to colocalize with CD14 in murine macrophage-like J774 cells, and their silencing reduced LPS-dependent production of TNF-α and CCL5/RANTES [145]. In Arf6-deficient Raw264.7 cells, the LPS-induced activation of NF-κB was lower than in their wild type counterparts suggesting a down-regulation of the MyD88-dependent signaling due to insufficient PI(4,5)P_2_ generation in the plasma membrane [73, 152]. Moreover, in these conditions, the cell surface level of TLR4 was also elevated and increased even further after stimulation of the cells with LPS likely due to an inhibited endocytosis of the receptor [152]. Furthermore, a depletion or inhibition of Arf6 blocked the TRIF-dependent signaling, which was reflected by a reduced phosphorylation of IRF3 and decreased expression of *Ifnβ1* [152, 153].

Recent studies have indicated that the endosomal signaling of TLR4 is negatively regulated by prostaglandin E2 in thioglycollate-elicited peritoneal murine macrophages. Inhibition of prostaglandin E2 receptor EP4 or inhibition of prostaglandin E2 synthesis enhanced the LPS-induced internalization of TLR4 and Syk activation, and consequently augmented the TRIF-dependent signaling [86]. Endocytosis of TLR4 is also negatively regulated by CD13 metallopeptidase [154]. CD13 associates with TLR4 in resting murine BMDM and DC and its expression is enhanced in LPS-stimulated cells. CD13 undergoes LPS-induced endocytosis together with TLR4 and CD14 and these proteins colocalizes in Rab5-positive endosomes. However, a knock-out of CD13 enhanced TLR4 endocytosis in macrophages and increased IRF3 activation and IFN-β production. Accordingly, mice lacking CD13 displayed enhanced IFN-β-triggered signaling leading to exacerbated ischemic muscle injury. The mechanism of the CD13 involvement in TLR4 endocytosis remains unknown, but it could be related to the raft localization of CD13 [154].

The LPS-induced internalization of TLR4 occurs via clathrin-independent and/or clathrin-dependent pathways, while *E. coli* bacteria undergo phagocytosis in which TLR4 is involved [3, 33, 41, 155, 156]. The TLR4 endocytosis depends on dynamin, a GTPase responsible for pinching off of clathrin-coated buds, some macro- and micropinosomes, and phagosomes [157, 158] inhibition of dynamin with dynasore prevented LPS-induced internalization of TLR4 in several cell types, including macrophages [40, 141, 155, 159]. Accordingly, in such conditions, the TRIF-dependent signaling was inhibited [40, 74, 95, 155, 159–161]. In addition, dynasore attenuated the LPS-induced cleavage of caspase-3 in murine microglia and almost completely abolished expression of IL-1β in these cells and in rat astrocytes overexpressing TLR4 [156, 159]. In HEK293 cells expressing TLR4/CD14 and a dominant negative dynamin II mutant (Dyn K44A), the NF-κB activation was upregulated in comparison to cells overexpressing wild-type dynamin, suggesting an upregulation of the MyD88-dependent signaling [41].

In contrast to the well-defined role of dynamin in LPS-induced TLR4 internalization, the role of clathrin in this process is more puzzling. Early electron microscopy studies suggested that endocytosis of LPS and CD14 is mainly clathrin-independent, yet no direct observation of TLR4 was possible at that time [162, 163] (see below). On the other hand, at least partial colocalization of TLR4 with transferrin, a typical cargo of clathrin-coated vesicles, was observed in HEK293 cells and in Ba/F3 cells transfected with TLR4/MD-2 and CD14 and stimulated with LPS for 45–60 min [41, 94]. In addition, partial colocalization of TLR4 with clathrin at the plasma membrane was observed in the HEK293 transfectants and in U373 glioma cells transfected with TLR4 and CD14 [164]. The authors claimed that in the spots of TLR4 that were positive for clathrin also CD14 was accumulated; however, it has to be noticed that they did not label CD14 directly but only observed Cy5-labeled LPS. In addition, in all the studies cited above CD14 and TLR4/MD-2 transfectants were used, which warrants their cautious interpretation in view of the relatively low level of TLR4 expression in native macrophages. Nevertheless, a line of studies using inhibitors of clathrin-dependent endocytosis support its contribution to TLR4 internalization. Monodansylcadaverine and chlorpromazine inhibited the LPS-induced internalization of TLR4 in Raw264.7 cells and in rat astrocytes overexpressing TLR4 [155, 159, 165]. Chlorpromazine also reduced production of CCL5/RANTES and IFN-β in those cells, and of IFN-β in murine DC and macrophages [155, 159, 165]. Similarly, incubation of J774 cells with another clathrin inhibitor, pitstop-2, reduced LPS-induced TNF-α and CCL5/RANTES production; however, the effect was weaker than that of dynasore [160]. Similarly, silencing of raftlin, a protein interacting with the heavy chain of clathrin and with clathrin-related adaptor protein AP-2 also reduced TLR4 internalization and negatively regulated the TRIF-dependent signaling pathway in human and murine cells poor in CD14 [165]. This additionally proves an engagement of clathrin in the internalization of TLR4 and points to the role of raftlin in this process [165].

Recent studies have revealed that disabled-2 (Dab2), an adaptor protein involved in the recruitment of some transmembrane receptors, e.g., low-density lipoprotein receptor, to clathrin-coated pits can also affect the TLR4 internalization [166]. Silencing of Dab2 in Raw264.7 cells increased the surface level of TLR4 in unstimulated cells but accelerated its LPS-induced endocytosis. In accordance, phosphorylation of IRF3 and expression of TRIF-dependent genes, including *Cxcl10, Ifit1, Ccl5* and *Ifnβ1*, was upregulated in these cells. Since TLR4 lacks a binding site for Dab2, the authors postulated that at steady state Dab2 binds and sequesters clathrin, thereby preventing TLR4 endocytosis. Stimulation of cells with LPS induces phosphorylation of Dab2 probably causing its dissociation from the plasma membrane and the following release of clathrin, and thereby allowing the clathrin-dependent internalization of TLR4. Thus, Dab2 can act as a negative regulator of TLR4 internalization [166].

Finally, the difference in the regulation of TLR4 endocytosis between murine macrophages and DC deserves attention. It was mentioned above that murine DC but not macrophages can phagocyte *E. coli* without a CD14 involvement [33]. Additionally, due to the lower level of CD14 in DC than in macrophages only in DC the endocytosis of TLR4 is facilitated by CD11b integrin [125]. CD11b in DC promotes both the MyD88-dependent and TRIF-dependent signaling of TLR4 in addition to its engagement in the receptor endocytosis. CD11b is also crucial for TLR4-triggered activation of the adaptive immunity by T lymphocyte [125]. The difference in the responses to LPS between macrophages and DC can be even more pronounced in humans. Only one human conventional (myeloid) DC subtype, cDC2 (also called CD1C +), expresses *TLR4* among them only subsets CD5^low/high^ expresses *CD14*, whereas murine conventional DC express both *Tlr4* and *Cd14* [29, 124, 167–172]. In addition, in human DC and also monocytes, TLR4 seems to be mainly intracellular [95, 173]. This suggests that in human DC, details of CD14-TLR4 signaling and trafficking can differ from those in their murine counterparts or in human macrophages. In addition, recent studies of murine mast cells have revealed that TLR4 trafficking in these cells can be regulated by huntingtin [174].

In summary, although many gaps in the understanding of the mechanisms controling TLR4 endocytosis remain, the crucial roles of CD14, PIs turnover, and dynamin in this process have been firmly established. Notably, modulation of the duration of the TLR4 presence at the plasma membrane after binding of LPS can affect the duration of the MyD88-dependent signaling and reciprocally also the activation of the TRIF-dependent endosomal pathway. The TLR4-triggered signaling is also modulated by the mechanisms controlling the intracellular trafficking of the receptor.

## Intracellular trafficking of TLR4: an overview

**Fig. 2:**
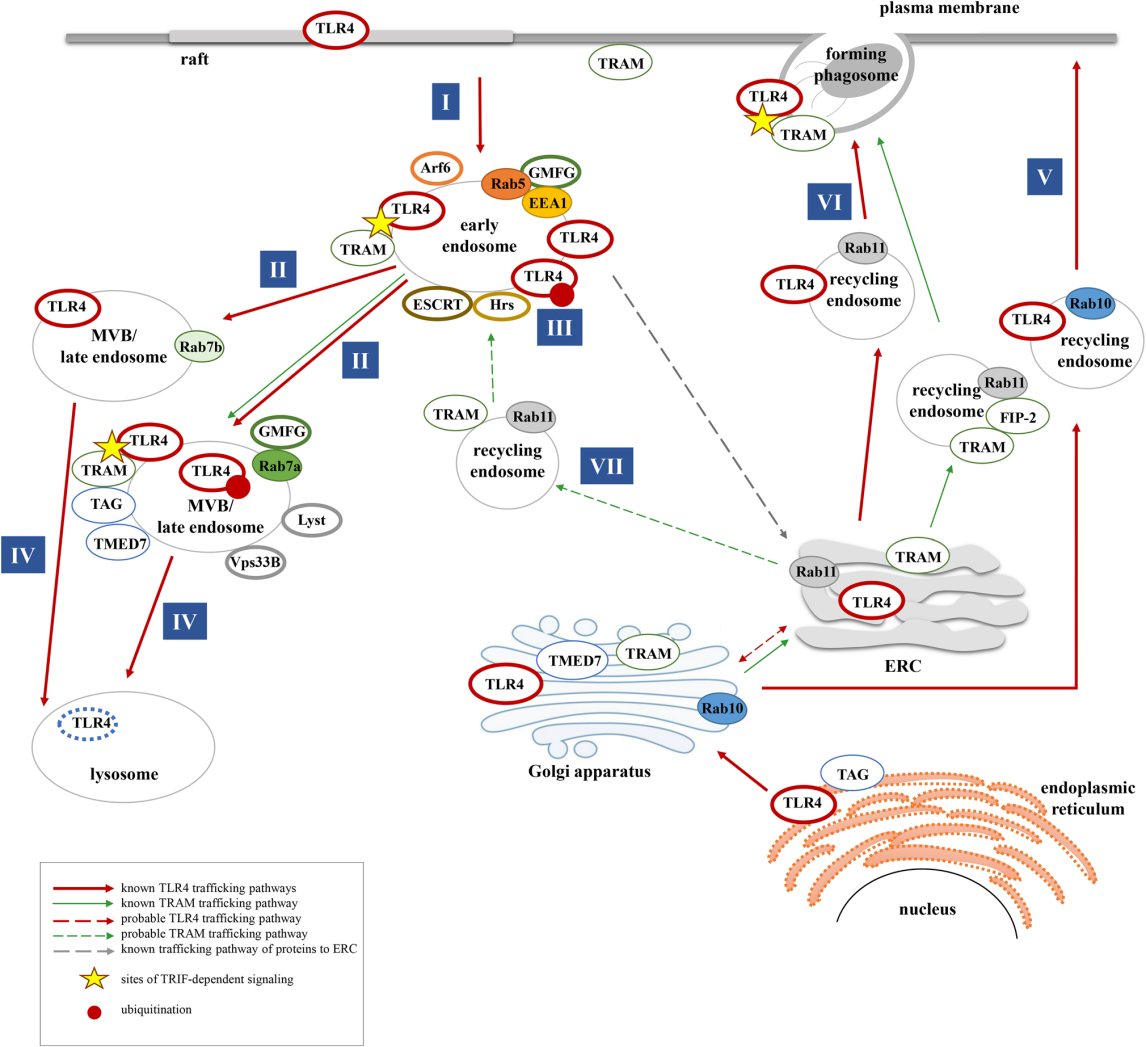
Intracellular trafficking pathways of TLR4 and TRAM. In unstimulated cells, TLR4 is present in the plasma membrane, Golgi apparatus and the ERC, whereas its adaptor protein TRAM is located in the ensosomes, the plasma membrane and Golgi apparatus. (I) After activation by LPS, TLR4 is internalized and translocates to Rab5- and EEA1-positive early endosomes where it interacts with TRAM and TRIF and initiates the TRIF-dependent signaling. (II) The association of TRAM with TLR4 and the activation of the TRIF-dependent pathway is sustained during maturation of early endosomes to Rab7a- or Rab7b-positive late endosomes. In late endosomes TMED7 and TAG disrupt the TRIF—TRAM interaction and facilitate termination of the signaling. (III) In early endosomes, Hrs and ESCRT recognize ubiquitinated TLR4 and sort it for degradation in late endosomes/MVB and lysosomes. (IV) TLR4 degradation and signaling can be regulated by proteins involved in the functioning of the endo-lysosomal compartment, like Lyst, GMFG, Rab7a and Rab7b, and Vps33B. (V) Upon stimulation with LPS, the intracellular TLR4 originally associated with the Golgi apparatus is transported via Rab10-positive endosomes to the plasma membrane. (VI) The intracellular pool of TLR4 derived from the ERC can trigger the TRIF-dependent signaling at the phagosome membrane. Both TLR4 and TRAM are transported to the phagosome membrane with a contribution from Rab11. (VII) Possible intracellular transport of TRAM in Rab11-positive vesicles to the early endosomes. It is unknown whether the well-established protein trafficking pathway from the early endosomes to the Golgi apparatus (dashed gray arrow) can be used by TLR4

TLR4 is synthetized in the endoplasmic reticulum where gp96, a paralog of Hsp90, is involved in proper folding of the receptor, while PRAT4A participates in its maturation (glycosylation). These proteins control folding and maturation of all TLRs [175, 176]. The newly synthetized TLR4 is transported to the Golgi apparatus, where it associates with MD-2; potentially TLR4 can reach the cell surface without an MD-2 assistance and then bind secreted MD-2 [177, 178]. In unstimulated cells, TLR4 can be detected not only in the endoplasmic reticulum, Golgi apparatus and the plasma membrane but also in Rab11-positive ERC [92, 94, 95, 179]. Latz et al. showed that in HEK293 cells transfected with TLR4 and MD-2, the receptor cycled between the Golgi apparatus and the plasma membrane. This suggested that the TLR4/MD-2 complex is internalized and directed to the Golgi apparatus to be transported back to the plasma membrane [92]. Later studies indicated that LPS binding changes the intracellular trafficking of TLR4 and revealed the complexity of this process. Thus, LPS-activated TLR4 is internalized and thereby its amount at the cell surface decreases with the duration of the stimulation [17, 40, 141]. TLR4 internalized by murine immortalized BMDM stimulated with 1 μg/ml LPS is first (after ~ 10 min of stimulation) detected in submembrane Rab5-positive vesicles related to early endosomes (Fig. 2, I) [180]. In murine microglia (BV2 cells), the TLR4 translocation to the Rab5-positive vesicles seems to be slower than in murine macrophages since a colocalization of TLR4 and Rab5 was visible only after 2 h of stimulation with the same LPS concentration [156], suggesting that the dynamics of TLR4 endocytosis is cell type specific. In the Rab5-positive endosomes TLR4 interacts with TRAM and activates the TRIF-dependent signaling cascade leading to the production of type I IFN and expression of IFN-induced genes. Next (after ~ 30–60 min of stimulation of immortalized BMDM) TLR4 is found in the membrane of Rab7-positive endosomes identified as late endosomes, where it also colocalizes with TRAM (Fig. 2, II) [180]. The endosomes marked by Rab7a or Rab7b are multifunctional in the context of TLR4 activity as the TRIF-dependent signaling can be maintained in some of them while others are sites of TLR4 degradation that progresses in lysosomes [94, 181–184], as discussed in more detail in the section below.

Ubiquitination is likely a signal targeting TLR4 for degradation and it increases significantly after stimulation of cells with LPS, but the exact type of this modification has not been established [41]. Ubiquitinated TLR4 is recognized by hepatocyte growth factor regulated tyrosine kinase substrate (Hrs), an early endosome-associated component of the ESCRT-0 complex which, together with other ESCRT complexes, causes clustering of the receptors and targets them to intraluminal vesicles in multivesicular bodies (MVB) and consequently to lysosomal degradation (Fig. 2, III and IV) [41].

Beside the Rab5- and Rab7-positive endo-lysosome compartment, in Raw264.7 cells, LPS-activated TLR4 (~ 10–20 min) is also found in Rab10-positive vesicles identified as a subtype of recycling endosomes, while prior to the stimulation TLR4 is located to the Golgi apparatus where also Rab10 can be detected (Fig. 2, V). Silencing of Rab10 decreased the amount of TLR4 in the plasma membrane and diminished the activation of both the MyD88- and the TRIF-dependent signaling pathway. It has been, therefore, proposed that Rab10 controls the replenishing of the plasma membrane pool of LPS-activated TLR4 from its reservoir in the Golgi apparatus [179]. Additionally, recent data have indicated that in J774 cells stimulated with LPS the plasma membrane pool of TLR4 can be replenished by de novo synthetized protein as well as by recycling of previously internalized receptor. The recycling route of TLR4 probably engages the ERC and ERC-related vesicles in which internalized TLR4 is transported back to the plasma membrane. This process depends on the plasma membrane-localized SNARE proteins syntaxin 11 and its partner SNAP-23 which can regulate fusion of the TLR4-bearing vesicles with the plasma membrane. A knock-down of syntaxin 11 or SNAP-23 suppressed the LPS-induced transport of TLR4 to the cell surface and promoted its lysosomal degradation [185]. Recycling of TLR4 can be down-regulated by TRPM7, as suggested by Granucci’s analysis of the TLR4 reappearance on the surface of TRPM7-depleted and LPS-stimulated macrophages [186].

Finally, unusual Rab11-dependent trafficking of an intracellular pool of TLR4 bypassing its plasma membrane localization was detected during phagocytosis of *E. coli* by human monocytes (Fig. 2, VI) [95, 187]. Rab11-positive vesicles also carry TRAM to the phagosomes, while in LPS-stimulated cells they participate in transporting GOLD domain-containing proteins to TLR4/LPS-harboring endosomes, thereby affecting the duration of the TRIF-dependent signaling [181, 182]. Thus, the Rab11-positive compartment plays an important role in the regulation of the TRIF-dependent signaling pathway of TLR4 discussed in detail in the following section.

The data presented above indicate that activation of the TRIF-dependent signaling cascade is coupled with the trafficking of TLR4 through endo-lysosomal compartments. Therefore, the process of transformation of early endosomes into late endosomes and lysosomes, which is correlated with changes of the protein and lipid composition of their membrane, acidification of the lumen and enrichment in hydrolytic enzymes, is crucial for both the signaling and the degradation of TLR4.

## Regulation of TLR4 signaling in endo-lysosomal compartment

Progressive acidification of the endosome interior provides an optimal environment for the dissociation of ligands from their receptors and for the activity of hydrolytic enzymes. In agreement, acidification of endosomes was found to induce splitting of TLR4/MD-2 dimers and dissociation of LPS from the receptor [188, 189], albeit the pH optimum for those processes has not been determined. In general, activation of endosomal proteases facilitates degradation of the internalized proteins, but for endosomal TLRs a limited proteolysis catalyzed by cathepsins and asparaginyl endopeptidase is necessary for their dimerization and signal transduction [152, 190]. Notably, TLR4 does not undergo such modification and binding of LPS overcomes the repulsion of two receptor ectodomains and forces TLR4 dimerization [69]. The acidic pH of endosomes could facilitate induction of the TRIF-dependent signaling by TLR4. Such hypothesis has been put forward by Ganglioff and co-workers based on their analysis of the crystallographic structure of TLR3, a receptor residing in endosomes and triggering the TRAM/TRIF-dependent signaling pathway. By analogy with TLR3, the acidic environment of endosomes (pH 5.5) would induce changes in the position of TLR4 ectodomains which after internalization face the endosome lumen. Concomitant conformational changes of the transmembrane and cytosolic regions of the receptor together with specific features of the endosome membrane, such as its high curvature and the presence of distinct species of PIs, could facilitate recruitment of TRAM to TLR4 [191].

Two approaches have been used to examine the role of endosome acidification in LPS-induced signaling of TLR4. Treatment of Raw264.7 cells with chloroquine, which neutralized the pH of the endosome interior, enhanced the depletion of LPS-activated TLR4 from the cell surface [155]. In agreement, chloroquine was shown to induce intracellular accumulation of LPS-activated TLR4 instead of its degradation, thus the stability of the TLR4/MD-2 complex was also prolonged [41, 189]. In chloroquine-treated Raw264.7 cells, the production of the MyD88- and TRIF-dependent cytokines was decreased, which was interpreted as a result of impaired recycling of TLR4 from endosomes to the plasma membrane [155]. On the other hand, knock-down of a ATP6V0D2, a subunit of the V-ATPase responsible for acidification of endosomes, attenuated the TRIF-dependent signaling pathway in Raw264 cells as reflected by a decreased expression of *Ifnb1* and upregulated expression of the MyD88-dependent *Tnfa, Il6 *and *Il12p40* [152]. In this case, the enhancement of the MyD88-dependent signaling and the reduction of the TRIF-dependent one correlated with reduced endocytosis of TLR4 and its prolonged maintenance on the cell surface. At the basis of this effect was an impaired interaction of the V-ATPase complex with Arf6 caused by the silencing of ATP6V0D2 [152], indicating that the regulation of TLR4 signaling via V-ATPase goes beyond controlling the endosome acidification and also involves its influence on the Arf6 activity. Ample data indicate that several other proteins which determine the functionality of the endo-lysosomal compartment, such as Lyst, Vps33B, GMFG and Rab7b, affect the TLR4-triggered signaling and the receptor degradation (Table 1).

Lyst is a lysosomal trafficking regulator contributing to endo-lysosomal biogenesis and also takes part in terminal maturation of secretory vesicles [192]. Structurally, Lyst belongs to BEACH domain-containing proteins which are large scaffolding proteins associated with cellular membranes due to the interaction of their PH domain with phospholipids, mainly PIs [193]. In murine BMDM and bone marrow-derived DC (BMDC) bearing a mutation in the *Lyst* gene leading to the production of a dysfunctional Lyst, neither the LPS-induced MyD88-dependent activation of MAP kinases and IκB nor the TLR4 endocytosis were affected; however, the TRIF-dependent IRF3 phosphorylation and its translocation to the nucleus were impaired. In agreement, that deleterious mutation of *Lyst* decreased the production of IFN-β, TNFα, and IL-12. Similar effects were observed in THP-1 cells of human origin. In vivo studies of pulmonary inflammation in mice showed that the Lyst dysfunction led to a lower production of TNF-α and IFN-β after administration of LPS, and protected the animals from endotoxin-induced lethal shock. This suggests that the dysfunction of the endo-lysosomal compartment observed in cells expressing the mutated form of Lyst inhibited the TRIF-dependent signaling. One can only speculate whether this impairment resulted from a faster degradation of TLR4 or an inefficient formation of its endosomal signaling complex. Later studies on the phagocytosis of *E. coli* and LPS-coated beads by BMDC expressing the mutated Lyst indicated that the recruitment of Rab7 (also called Rab7a) to maturing phagosomes, but not their acidification, was affected [183].

Vacuolar protein sorting-associated protein (Vps) 33B, a homolog of yeast Vps33p, is another factor regulating TLR4 signaling and degradation. Mammalian Vps33B and closely related Vps33A are soluble proteins of the Sec1/Munc18 family which, together with SNAREs, allow fusion of intracellular compartments with lysosomes. Vps33B interacts with the VIRAP protein and with Rab10, Rab11, and Rab25 [194, 195]. After silencing of Vps33B in murine macrophages, the phagosomal clearance of *E. coli* was impaired even though the rate of phagocytosis was not changed. Furthermore, the trafficking of LPS-activated TLR4 to Rab5- and further to Rab7-positive endosomes was also similar to that in wild-type cells. In both Vps33B-depleted and control macrophages, TLR4 was also present in Rab11-positive endosomes. However, degradation of LPS-activated TLR4 was impaired after Vps33B depletion and the receptor accumulated in LPS-stimulated cells, which resulted in an overproduction of cytokines such as IL-6, TNF-α and IFN-β. Collectively, these data suggest that Vps33B has an important function in the maturation of phagosomes and also in a late step of endosome maturation, i.e., the fusion with lysosomes. Therefore, Vsp33B can be viewed as a negative regulator of the TLR4-induced pro-inflammatory response [180].

Glia maturation factor-γ (GMFG) is a newly discovered actin depolymerization factor which binds the Arp2/3 complex and inhibits nucleation of actin monomers and also promotes debranching of actin filaments, processes important for endosome trafficking. GMFG is expressed mainly in immune cells and microvascular endothelial cells, where it facilitates recycling of internalized β1-integrin [196]. GMFG also affects the internalization and trafficking of LPS-activated TLR4 and thereby the TLR4-induced pro-inflammatory responses. In THP-1 cells, GMFG associates with early and late endosome markers, including EEA1, Rab5, and Rab7. Depletion of GMFG in those cells and in primary human macrophages resulted in a delayed internalization of TLR4 and its abnormal accumulation in the plasma membrane and Rab5-positive vesicles. This retention of TLR4 in the plasma membrane and in early endosomes significantly enhanced the LPS-induced activation of NF-κB, MAP kinases and IRF3, and the production of TNF-α, IL-6 and IFN-β at both the mRNA and protein level. In accordance, GMFG overexpression decreased the production of pro-inflammatory cytokines. Interestingly, production of the anti-inflammatory IL-10 was slightly increased in these conditions [196].

Rab7b is expressed selectively in some cell types, e.g., in monocytes and monocyte-derived immature DC, and is involved in the transport of cargo to late endosomes and lysosomes [184, 197, 198]. Silencing of Rab7b in Raw264.7 cells increased the overall level of TLR4, including its cell-surface pool [184]. More-detailed studies on TLR4 localization in EEA1- and LAPM-1-positive compartments indicated that silencing of Rab7b caused accumulation of TLR4 in early endosomes, while overexpression of Rab7b led to an accelerated TLR4 translocation to late endosomes/lysosomes. Those data suggested that Rab7b is required for lysosomal degradation of TLR4. Indeed, overexpression of Rab7b in peritoneal macrophages and Raw264.7 cells decreased TLR4 protein level without affecting its mRNA level [184]. An increased expression of Rab7b also down-regulated TLR4 surface level in human DC treated with a water-soluble fraction of the worm *Trichuris suis* [199]*.* In addition, studies on the cerebral ischemic stroke in the rat revealed an increased expression of Rab7b in the brain after the stroke. In turn, overexpression of Rab7b in rat brain reduced the TLR4 and NF-κB levels following the stroke and also reduced the production of pro-inflammatory cytokines in both signaling cascades of the receptor. Thus, the overexpression of Rab7b down-regulated the activation of MAP kinases, NF-κB and IRF3 as well as inhibited the production of IL-6 and IFN-β in the rat brain [200]. Also in other studies, overexpression of Rab7b reduced the TLR4-triggered cytokine release, including that induced by a water-soluble fraction of *T. suis* in human DC [199]. It should be emphasized, however, that the overexpression of Rab7b led to a down-regulation of NF-κB level in addition to that of TLR4, suggesting that also this effect contributed to the reduction of the TLR4-triggered pro-inflammatory responses [199, 200].

Rab7a and Rab7b exhibit 65% sequence similarity [197] and have partially overlapping cellular localizations and functions in maturation of endosomes, yet the two proteins regulate TLR4 signaling differently. Rab7a is required for the activation of the TRIF-dependent signaling cascade in LPS-stimulated BMDMs and does not interfere with the MyD88-dependent one. Also during phagocytosis of LPS-coated beads, Rab7a-positive phagosomes recruited TBK1, the kinase responsible for phosphorylation of IRF3 [183]. Recent studies have indicated that both Rab7a and Rab7b can be involved in retrograde transport of cargo from late endosomes to the *trans*-Golgi network and called into question the involvement of Rab7b in the transport of selected proteins from early to late endosomes [198, 201]. The retrograde transport is mediated by a multiprotein complex called retromer which retrieves membrane-bound receptors, e.g., cation-independent mannose-6-phosphate receptor, from endosomes and transports them to the *trans*-Golgi network and to the plasma membrane. The core of the retromer is formed by the heterotrimer of Vps35, 29 and 26. Silencing of Vps35 in microglia increased their inflammatory response to LPS, which was linked with an impaired trafficking of Trem2, an immunomodulatory microglial surface receptor [202]. Taking into account that Rab7a, Rab7b, and Vps35 alter the LPS-induced inflammatory responses it seems plausible that retrograde transport can be important for the recycling/retrieval of TLR4, but further studies are required to confirm this hypothesis.

Maturation of endosomes is linked with changes of the lipid composition of their membrane. Our studies also indicated that such changes are important for the regulation of the intensity of TLR4-dependent pro-inflammatory responses. We found that accumulation of lysobisphosphatidic acid, a phospholipid enriched in late endosomes/lysosomes, strongly inhibited the TRIF-dependent signaling cascade and decreased LPS-dependent CCL5/RANTES production [203].

Taken together, the data discussed above indicate that disturbances in the endocytosis of LPS-activated TLR4 and also dysfunctions of the endosomal/lysosomal machinery ultimately affect TLR4-triggered pro-inflammatory responses. A proper maturation of endosomes is necessary for the TRIF-dependent signaling to occur and can also affect the MyD88-dependent one, while slowing down of the TLR4 degradation increases the pro-inflammatory response. Whether a retrograde transport of TLR4 from Rab7-positvie endosomes via the Golgi to the plasma membrane, which could sustain activation of the receptor by extracellular LPS, is a physiological phenomenon remains an intriguing open question.

## Rab11-positive recycling endosomes in LPS-induced trafficking of TLR4 and TRAM

Early endosomes identified by Rab5 presence provide a sorting environment for diverse internalized membrane proteins. Some of them recycle back to the *trans*-Golgi network or to the plasma membrane, others are directed to lysosomes for degradation. In general, the routes of protein recycling to the plasma membrane are classified as “fast” and “slow”. Fast recycling involves Rab4- or Rab35-marked vesicles which bud off of the early endosomes and next fuse with the plasma membrane [204]. On the other hand, proteins retrieved by the slow recycling route are delivered from early endosomes to the Rab11-positive ERC closely opposing the Golgi apparatus, and then are transported back to the plasma membrane by Rab11- and EHD1- or Arf6-positive recycling endosomes [153, 204, 205]. Rab11-poisitive vesicles participate in the trafficking of the archetypical recycling protein, the transferrin receptor [206].

Rab11-positive compartments are also crucial for the TLR4 trafficking and activation during phagocytosis of Gram-negative bacteria. It was found that Rab11-bearing ERC is a source of TLR4 that is transported in a Rab11-dependent manner toward forming phagosomes (Fig. 2, VI). This intracellular pool of TLR4 omits the plasma membrane and triggers the TRIF-dependent signaling at the phagosome membrane. TRAM, required at the onset of this signaling pathway, is also transported to the phagosome membrane with a contribution from Rab11. Local accumulation of IRF3 and production of IFN-β follow [95]. Further studies have revealed that FIP-2, a Rab11 effector protein, is required for the TRAM recruitment to the forming phagosomes. Rab11, FIP-2, TRAM, and TRIF assembly into a complex which is recruited to the TIR domain of activated TLR4. FIP-2 guides the TRAM accumulation at the forming phagosomes to activate Rac1 and Cdc42, thereby governing actin filament rearrangement and the uptake of the bacterium, and also to induce the TRAM/TRIF-dependent signaling of TLR4 [187].

Rab11-positive recycling endosomes can also be involved in the intracellular trafficking of TLR4 during stimulation of cells with LPS. Indeed, a FRAP analysis of TLR4 mobility in LPS-stimulated cells revealed its high dynamics suggesting that TLR4 enters and exits the ERC [164]. Moreover, silencing of Rab11 or FIP-2 inhibited the TRIF-dependent signaling of TLR4 triggered by LPS, resembling the effects seen during phagocytosis [95, 187]. At present there are no other data showing that during stimulation of cells with LPS Rab11-positive vesicles can transport TLR4 from its intracellular reservoir directly to endosomes. More likely is a contribution of Rab11 to the transport of TRAM toward the endosomes which acquired LPS-TLR4 from the plasma membrane following induction of the MyD88-dependent signaling cascade. Rab11 can determine the localization of TRAM in unstimulated cells. Overexpression of a Rab11 in HEK293 cells expressing also TLR4, MD-2, CD14, and TRAM resulted in accumulation of TRAM in the ERC, which was concomitant with its depletion in the Golgi apparatus [164]. As mentioned above, the pool of TRAM involved in the endosomal TLR4 signaling does not traffic to endosomes from the plasma membrane [40, 182]; therefore, its possible sources include the Golgi apparatus and the Rab11-positive ERC (Fig. 2, VII). TRAM can be accumulated in the ERC with the help of Arf6-dependent transport from the vicinity of the plasma membrane [153]. Upon a subsequent stimulation of cells with LPS, the TRAM-Rab11-FIP-2-TRIF complex can be transported from the ERC to LPS/TLR4-harboring endosomes where the TRIF-dependent signaling is triggered [95, 164]. This Rab11-dependent trafficking of TRAM could facilitate activation of the endosomal signaling pathway of TLR4. Expression of a dominant negative or constitutively active Arf6 variant interfered with the endosomal TLR4 signaling indicating its dependence on the Arf6-mediated intracellular TRAM trafficking [153].

Rab11 is also involved in the delivery to endosomes of two other proteins—transmembrane emp24 domain-containing protein (TMED) 7 and TRAM adaptor with GOLD domain (TAG), both involved in the TRIF-dependent signaling of TLR4. TMED7 and TAG cooperate to promote termination of TLR4 signaling. Silencing of TAG or TMED7 resulted in upregulation of the TRIF-dependent signaling reflected by an increase of the ISRE reporter gene expression and enhanced production of CCL5/RANTES. Moreover, in cells with a reduced level of TAG or TMED7 degradation of TLR4 was inhibited [181, 182]. At the basis of the regulatory function of TMED7 and TAG in TLR4-triggered signaling is their negative influence on the stability of the TRAM/TRIF complex. These two proteins contain a GOLD domain allowing their homotypic interaction [181, 182]. TAG, expressed exclusively in humans, is a splicing variant of TRAM and also carries the TIR domain [181]. Upon binding to the TIR domain of TRAM, TAG displaces TRIF from the TRAM/TRIF complex. In unstimulated cells, TAG localizes mainly to the endoplasmic reticulum while TMED7 is present in the Golgi apparatus. After LPS stimulation, the two proteins traffic to endosomes. The colocalization of TMED7 with TLR4 and TRAM begins in EEA1-positve early endosomes and culminates in Rab7-positive late endosomes where TMED7 facilitates disruption of the TRAM/TRIF complex by TAG, which eventually leads to degradation of TLR4 [181, 182].

The above data indicate that Rab11-positive compartments are involved in the LPS-induced trafficking of TRAM and also of other proteins regulating the LPS-induced TRAM/TRIF-dependent signaling. In the following sections, we discuss how the TLR4-triggered signaling can be affected by cellular trafficking of CD14 and internalization of LPS.

## Cellular trafficking of CD14 and its relation to the trafficking of TLR4

The participation of CD14 in the activation of TLR4 by LPS and in the endocytosis of LPS-activated TLR4 followed by the TRIF-dependent pro-inflammatory signaling is well established, as discussed above. Notably, CD14 is also involved in the internalization of high doses of LPS which engages scavenger receptors and leads to LPS detoxification by J774 cells [160], as described in the following section. However, it has to be emphasized that apart from LPS, CD14 binds a broad spectrum of other molecules, including PAMPs, like peptidoglycan, lipoteichoic acid, CpG DNA, and phospholipids [51, 207], and also DAMPs such as β-amyloid [208]. Thus, CD14 can affect the activity of several PRRs. The binding of phospholipids by CD14 is interesting in the context of the LPS-induced pro-inflammatory signaling of TLR4. Among the phospholipids bound by CD14 are 1-palmitoyl-2-glutaroyl-*sn*-glycero-3-phosphorylcholine and 1-palmitoyl-2-(5-oxovaleroyl)-*sn*-glycero-3-phosphorylcholine, two forms of oxidized 1-palmitoyl-2-arachidonyl-*sn*-glycero-3- phosphorylcholine (oxPAPC) released from dying cells at the site of tissue injury. The oxPAPC-induced internalization of CD14 depletes its cell surface pool and as a consequence makes these cells less sensitive to LPS. However, in LPS-primed murine macrophages and DC, the CD14-dependent delivery of oxPAPC into the cells leads to the activation of caspase-11 and caspase-1 followed by IL-1β release. Notably, oxPAPC did not induce pyroptosis of such cells leaving them hyperactive to produce IL-1β without the lethal outcome of an inflammatory response and sepsis in mice [144, 209]. These data support the TAXI name given to CD14 as it is able to capture various cargo in addition to TLR4/MD-2/LPS and deliver them to signaling-competent locations [17].

Only scarce data allow speculation on the pathway(s) involved in the internalization of CD14 carrying LPS and other ligands, like oxPAPC, and also in the constitutivie endocytosis of CD14 in resting murine macrophages [17]. The latter is of importance since a down-regulation the cell surace level of CD14 and TLR4 in resting cells should prevent excessive LPS-induced pro-inflammatory signaling of TLR4 [161, 210]. This mechanism can also contribute to the antagonistic activity of *R. spheroides* LPS toward human and murine TLR4. Studies performed on murine BMDM showed that this LPS species induced CD14 endocytosis preventing subsequent binding of the pro-inflammatory *E. coli* LPS to CD14 and consequently to TLR4. The ability of *R. spheroides* LPS to induce CD14 endocytosis was determined by diphosphorylation of its lipid A [17].

The LPS-induced endocytosis of CD14 is independent of the signaling activity of TLR4, but it was reduced by a Syk inhibitor or a knocdown of PLCγ2 [33]. This suggests that, upon LPS binding, CD14 and TLR4 are internalized by the same route discussed above for TLR4 endocytosis. Since this LPS-induced uptake of CD14/TLR4/MD-2 does not require the signaling activity of TLR4, it has been proposed to be controled by CD14 itself [17]. However, it is still unknown whether the endocytic pathway triggered by LPS is the same as that involved in the constitutive internalization of CD14 in unstimulated cells or in the CD14-mediated uptake of other ligands.

Studies on the endocytosis of other GPI-anchored proteins (GPI-APs) may shed light on the LPS-independent internalization of CD14. The majority of GPI-APs are internalized via the dynamin- and clathrin-independent carrier (CLIC) endocytic pathway which also contributes markedly to fluid phase uptake. The internalization of CD14 detected in resting J774 cells displayed properties of a CLIC-mediated uptake; however, the apparent similarities between the CD14 and TLR4 uptake found in those inhibitor-based studies do not allow unequivocal identification of the endocytic pathway involved [161]. The CLIC-mediated endocytosis is sensitive to actin depolymerization and requires activation of the Arf1 and Cdc42 GTPases [211]. Cdc42 interacts with PS in the inner leaflet of the plasma membrane. Depletion of CDC50, the α-subunit of the flippase complex, and the following inhibition of PS transport from the outer to the inner monolayer of the plasma membrane increased the surface level of CD14 in unstimulated THP-1 macrophages [212]. These data suggest that CD14 can be internalized by CLIC-mediated endocytosis involving Cdc42 and PS. An attempt has been undertaken to assess whether the CLIC-mediated endocytosis is also involved in the internalization of CD14 upon LPS binding. It was found that, depletion of galectin-3, a protein triggering CLIC-mediated endocytosis via clustering of cell-surface glycosylated proteins with glycosphingolipids, did not affect the LPS-induced internalization of either CD14 or TLR4 [17, 213]. Those results spoke against a role of the galectin-3-facilitated endocytosis of CD14 in LPS-stimulated cells; however, a hypothetical involvement of galectin-3-independent CLIC-mediated pathway(s) cannot be ruled out.

Internalized GPI-APs accumulate in early endosomes which therfore earn the name of GPI-AP-enriched early endosomal compartmet (GEEC). The GEEC undergoes fast but only modest acidification to pH ~ 6, which is above the average pH of other early endosomes. The acidic pH of GEEC can facilitate the release of ligands from the internalized GPI-APs [211, 214]. Inhibition of V-ATPase reduced the CLIC/GEEC endocytosis of dextran, a fluid phase marker [211]. As mentioned earlier, a knock-down of the ATP6V0D2 subunit of V-ATPase also impaired the LPS-induced endocytosis of TLR4 [152]. It remains to be established whether in those conditions, the inhibition of the internalization of TLR4 was a consequence of a disturbed uptake of CD14.

At present, there are no data indicating that upon LPS binding CD14 can recycle to the plasma membrane either via the fast Rab4/Rab35-dependent pathway, the slow one dependent on Rab11, or via Rab10-enriched recycling endosomes, as it was shown for TLR4 [179]. On the contrary, it has been established that at least a fraction of the internalized CD14 is degraded in lysosomes [17]. Even in cells not stimulated with LPS CD14 was detected in perinuclear structures and in MVB-like vesicles [163]. Stimulation of cells with LPS increased the degradation of CD14 in lysosomes while chloroqine treatment inhibited this process [17]. Notably, CD14 internalized by human monocytes during phagocytosis of *E. coli* or monosodium urate crystals (a sterile phagocytic stimulus) is cleaved in phagolysosomes by elastase and relased as presepsin [54] (see chapter “Signaling pathways triggered by TLR4”).

It is an open question whether disturbances in the endo-lysosome functioning caused, e.g., by mutation/knock-down/inhibition of endosomal proteins listed in Table 1 can affect the cellular level and localization of CD14 and thereby the LPS-induced TLR4 activation. This possibility is usually neglected in analyses of the impact of endo-lysosomal proteins on TLR4 trafficking and signaling.

## LPS uptake and detoxification

Clearance of LPS from circulation and its detoxification help resolve the inflammation induced by bacterial infection. These processes are also important for the removal of the gut microbiota-derived LPS which got access to the bloodstream [215]. There are several mechanisms of LPS inactivation in the human body acting both extracellularly and intracellularly. The latter follows LPS uptake by hepatocytes which remove LPS into the bile, and by immune cells capable of detoxifying the endotoxin enzymatically [216].

The extracellular inactivation of LPS is executed by several lipid A-neutralizing proteins which circulate in the bloodstream, like bactericidal permeability-increasing protein, lactoferrin, lysozyme, collectins, and also anti-LPS antibodies [217]. Also in the blood LPS can be bound by lipoproteins, such as chylomicrons and high, low, and very low-density lipoproteins (HDL, LDL, VLDL) to be next transported to the liver [218, 219]. The binding of LPS to the lipoproteins is catalyzed by phospholipid transfer protein, cholesteryl ester transfer protein, LBP, and sCD14. Liver is the main source of sCD14, also LBP is produced predominantly by the liver and in addition by epithelial cells of lungs and the gastrointestinal track [220]. LPB and sCD14 are both acute phase proteins upregulated significantly during sepsis [45, 221]. High concentrations of LBP attenuate TLR4 activation by inhibiting the transfer of LPS from CD14 to MD-2 while sCD14 can accept LPS form membrane-bound CD14 and accelerate its transfer to HDL and subsequent detoxification in the liver [49, 207, 215, 222, 223].

Liver also plays an essential direct role in the clearance of LPS from circulation and its excretion into the bile. In mice injected in the tail vein with 5 μg LPS, 90% of the endotoxin accumulated in the liver within 60 min [224]. The mechanism of the LPS detoxification in the liver is not fully clear but it is known to engage various types of hepatic cells, including liver sinusoidal endothelial cells, hepatocytes, and hepatic macrophages—the Kupffer cells. All these cells can internalize LPS, but mainly the macrophages inactivate the internalized endotoxin enzymatically [224–227]. If not internalized by hepatocytes directly, LPS is transferred to them from the other cells and is next secreted into the bile [224].

It is worth emphasizing that the mechanism of LPS endocytosis preceding its intracellular detoxification depends on its formulation which includes aggregates, OMV, lipoprotein-bound LPS, and LPS monomers. OMV and LPS-enriched liposomes are likely internalized in a clathrin-dependent and CD14-independent way by macrophage. The binding of LPS to lipoproteins affects the way it is detoxified in the liver. The endocytosis of HDL-LPS by Kupffer cells was not as effective as that of free LPS [224], indicating that the HDL-LPS complexes are rather internalized by cells highly expressing scavenger receptors, such as liver sinusoidal endothelial cells and hepatocytes [227]. The role of the scavenger receptors, e.g., CD36, CLA-1/SRB1, CLA-2 and SR-A of immune cells, in LPS-induced processes is more intricate and includes the CD14-dependent uptake of high doses of LPS but also the activation of immune responses [160].

It has been found recently that LPS can also be internalized in complex with secretoglobin 3A2 and HMGB1. The first protein is abundantly expressed in airway epithelial cells and is internalized via the SDC1 receptor. The endocytosis of HMGB1, in turn, is mediated by the RAGE receptor expressed in epithelial cells and macrophages. Both proteins facilitate leakage of LPS from endosomes to the cytosol where it is bound by pro-caspase-11 and activates the NLRP3 inflammasome [113, 228].

TLR4 makes a small contribution to the internalization of LPS by immune cells [229]. In agreement, blocking of TLR4 with a neutralizing anti-TLR4 antibody did not affect the internalization of a relatively high dose of LPS (1000 ng/ml) [160]. Although TLR4 did not directly participate in the uptake of high amounts of LPS by macrophages, it could indirectly reinforce its clearance from plasma by hepatocytes, as a knock-down of hepatocyte TLR4 inhibited this process [26]. It was also shown that TLR4, CD14, and MD-2 participate in the clustering of CD11b/CD18a integrins involved in LPS endocytosis in hepatocytes [230]. Moreover, TLR4 can regulate deacylation of LPS by immune cells. In TLR4-deficient mice, significantly less LPS was deacylated at the site of infection than in wild-type littermates [231]. In accordance, LPS and commensal Gram-negative bacteria increased the expression of acyloxyacyl hydrolase (AOHA) in macrophages and DC suggesting that activation of TLR4 can activate production of this enzyme [232, 233]. AOHA is a highly conserved hydrolase which recognizes LPS and removes secondary fatty acids at positions 3′, 2′ and/or 2 of lipid A, converting a hexa-acyl (also hepta- or penta-acyl) structure into one that has only four acyl chains. The resulting tetra-acyl LPS molecule binds to MD-2 but does not initiate TLR4 signaling and is also inert to caspase-based detection systems [234]. Similarly, weaker inflammatory responses are induced by LPS dephosphorylated by host phosphatases, like the intestinal alkaline phosphatase which participates in detoxification of LPS in the gut lumen [235]. Deacylation and dephosphorylation of LPS deprived it of a pro-inflammatory activity especially in human cells [59, 61], due to a weaker or no activation of human TLR4 by these LPS species, as discussed above. Notably, the vast majority of in vivo studies on LPS detoxification have been carried out on mice the TLR4 of which can be activated by a broader range of LPS species (see above), but which are much less sensitive to LPS-induced septic shock than humans [236].

Intestinal alkaline phosphatase is produced in the duodenum, whereas high expression of AOAH was found in the liver, mainly in Kupffer cells with a minor contribution of DC [235, 237]. AOHA expression was also detected in macrophages and DC from the large intestine and in lung macrophages and neutrophils [232]. When LPS was applied subcutaneously, mimicking its release from bacteria in a tissue other than blood, up to 70% of the LPS was deacylated by AOHA before it left the site of injection. This in situ detoxification of LPS is carried out by immune cells, including neutrophils, DC, and macrophages [231].

Deacylation of LPS is a slow process taking several days. Therefore, during infection, the activity of AOHA has little impact on the early response to LPS and cytokine production, but it is necessary to terminate the inflammation and affects the activation of B lymphocytes [231, 237]. Many aspects of AOHA activity are unresolved, the most intriguing one being whether the main side of its action are organelles of immune cells or the extracellular space [231]. In light of the data on the role of metabolic endotoxemia in the development of metabolic diseases, and a growing number of fatal sepsis cases worldwide, the mechanisms of endotoxin disposal by immune cells and overall by the human body deserve close attention.

## Contribution of abnormal trafficking of TLR4 and CD14 to pathogenesis of certain diseases

The multiple pathways of TLR4 trafficking in diverse cells require tight regulation to ensure an optimal magnitude and duration of pro-inflammatory responses induced by LPS and DAMPs. For this reason, several human hereditary and neurodegenerative diseases caused by disturbances in the functioning of the endo-lysosmal compartment are linked with TLR4 hyperactivation and inflammation contributing substantially to their pathogenesis.

This fatal relationship is exemplified by lysosomal storage disorders (LSD) caused by hereditary dysfunctions of lysosomes which are accompanied by an abnormal activation of TLR4 and production of pro-inflammatory cytokines [238, 239]. These responses can be triggered by an extracellular accumulation of undegraded substances which act as DAMPs and also by excessive TLR4 stability and persistent signaling caused by an impaired lysosomal proteolysis in cells of LSD patients. In particular, TLR4 has been shown to be involved in the development of mucopolysaccharidosis (MPS), a heterogenous group of LSD resulting from deficiencies in lysosomal enzymes degrading glycosaminoglycans (GAGs). Various MPS subtypes affect different tissues and organs, including central nervous system, bones, muscles, and the connective tissue [238]. Under physiological conditions, hyaluronian of the GAG family is transported to the extracellular matrix while other GAGs, like heparan sulphate, are exposed on the cell surface and remain bound to the plasma membrane via a proteoglycan core. Thereby heparan sulphate of epithelial cells affects the adhesion of immune cells during inflammation. Several GAGs also induce production of pro-inflammatory chemokines and cytokines. Studies conducted on animal models of MPS VI and/or VII showed an increased level of IL-1β and TNF-α and also an upregulated expression of several other inflammatory markers in synovial fluid, fibroblast-like synoviocytes and serum. Those changes were linked with TLR4 activity as a knock-down of TLR4 in MPS VII animals normalized the serum level of TNF-α and phosphorylation of chondrocyte STAT1 and also alleviated some of the pathological effects of the disease in bones and joints [240, 241]. A similar line of data indicated a contribution of TLR4-induced pro-inflammatory signaling of microglia to the onset of neurodegenerative MPS III [242]. The pro-inflammatory signaling of TLR4 is considered to be triggered by GAG—DAMPs which accumulate in the extracellular space. These include hyaluronian which stimulates TLR4 in cooperation with CD44, and also heparan sulphate and its derivatives resulting from an incomplete degradation of this GAG in lysosomes and subsequent exocytosis of its products. However, an increased level of TLR4 has also been detected in fibroblast-like synoviocytes of MPS VI animals. In addition, these cells have increased mRNA levels of LBP, CD14, and MyD88. The upregulation of TLR4 level was caused by its accumulation in dysfunctional lysosomes with an unchanged expression of the *Tlr4* gene [240, 241]. Collectively, these results indicate that an impaired lysosomal degradation of TLR4 contributes to the inflammation in MPS.

An increased activity of TLR4 is also linked with the pathogenesis of another LSD, Niemann–Pick's type C (NPC) disease leading to progressive neurological dysfunctions in children. NPC is caused by mutations in the *NPC1* or *NPC2* genes causing malfunctioning of the encoded proteins, which results in an aberrant endosomal membrane flow and accumulation of cholesterol and other lipids in endo-lysosomes. TLR4 level is increased in fibroblasts and glial cells of NPC patients and in *Npc1* knock-out mice, respectively, as a result of its impaired degradation. This leads to the accumulation of TLR4 in endosomes and upregulation of its signaling pathways enhancing the production of pro-inflammatory cytokines, such as IL-6, IL-8, and IFN-β. The increased inflammatory response, particularly the glial production of IL-6, can contribute to the pathogenesis of NPC via both the autocrine and paracrine effects of this cytokine [243].

A participation of the TLR4 receptor has also been noted in the development of cystic fibrosis (CF)—an autosomal recessive disorder caused by mutations in the *CFTR* gene encoding cystic fibrosis transmembrane conductance regulator, a chloride channel expressed in airway epithelial cells and, to a lower extent, also in other cells. The disease is manifested chiefly by a dysfunction of the airway epithelium, which leads to a dehydrated and acidic airway surface liquid favoring chronic bacterial infections [244, 245]. A characteristic feature of cystic fibrosis is an excessive inflammatory response leading to lung damage [244, 245]. Data concerning the role of bronchial epithelial cells in the inflammatory response in cystic fibrosis are inconsistent. Early studies reported a decreased or unchanged expression of TLR4 in those cells in comparison with the cells of healthy donors [244, 246]. In contrast, a recent study on immortalized bronchial epithelial cell derived from a CF patient showed an increased level of TLR4 on the cell surface and its impaired transport to lysosomes likely contributing to an enhanced inflammatory response to LPS [247]. However, the hyperinflammatory response in CF lungs is mainly attributed to macrophages. Accordingly, macrophages from CFTR knock-out mice had an elevated level of TLR4 compared with the wild type, with no differences in the amount of TLR4 mRNA [248]. The deletion of the *Cftr* gene caused accumulation of TLR4 in early endosomes in macrophages and compromised its degradation, thereby leading to an increased activation of NF-κB, MAPK, and IRF3. The retention of TLR4 in early endosomes could ensue from an aberrant maturation of endosomes in CFTR-depleted LPS-stimulated cells, insufficient endosomal acidification, or perturbations in cholesterol trafficking caused by the CFTR deficiency [248]. These mechanisms can also cause the accumulation of TLR4 and an excessive pro-inflammatory response to LPS of human macrophages ultimately leading to the lung disease of cystic fibrosis patients [248].

The arthrogryposis–renal dysfunction–cholestasis syndrome is a rare fatal disease caused by autosomal recessive mutations in the *VPS33B* gene. Characteristic symptoms of this disease comprise arthrogryposis, renal tubular acidosis, and neonatal cholestatic jaundice. The patients also suffer from persistent infections and sepsis [180, 249]. As it was described above, dysfunction of VPS33B leads to an impaired degradation of TLR4 and enhanced pro-inflammatory response of macrophages to LPS via the MyD88- and TRIF-dependent pathways. It has been proposed that such exaggerated TLR signaling causes so-called immune paralysis whereupon the immune system loses the ability to respond to secondary infection [180].

Studies on Chédiak–Higashi syndrome (CHS) have expanded the list of diseases linked with disturbances in TLR4 trafficking and signaling. CHS is a rare autosomal recessive disease characterized, among others, by partial albinism, neurological problems, and immunodeficiency. CHS patients suffer from persistent and recurrent infections of the skin, respiratory tract, and mucous membranes. Human CHS and its corresponding mouse disorder *beige* are caused by mutations of the *LYST* gene [192]. Recent studies indicate that Lyst controls endosomal TRIF-dependent signaling of TLR4, as described above. In mice, a loss of a functional Lyst impaired inflammatory responses against Gram-negative bacteria and enhanced bacterial dissemination in tissues but also lowered the susceptibility to LPS-induced septic shock as a result of a reduced production of pro-inflammatory cytokines [183].

TLR4 also participates in the development/progression of neurodegenerative disorders associated with Alzheimer's disease (AD), Parkinson's disease (PD), multiple sclerosis, and amyotrophic lateral sclerosis (ALS). Due to its engagement in neurodegeneration, TLR4 is considered a therapeutic target in the treatment of these diseases [250]. At the early stages of the development of neurodegenerative diseases, activation of TLR4 may be beneficial for the clearance of β-amyloid (AD) and α-synuclein (PD) and can prevent accumulation of their aggregates characteristic for these diseases. However, a prolonged overactivation of microglia may lead to the release of neurotoxic products and damage neurons [251, 252]. Such increased activity of microglia can result from their abnormally high content of TLR4. An increased expression of TLR4 was observed in brains of a murine AD model (overexpressing amyloid precursor protein) and in AD patients in areas surrounding plaques [251, 253]. Moreover, β-amyloid increases expression of TLR4 in cortical neurons, which further activates JNK and caspase-3 and contributes to neuron death [254]. An elevated expression of TLR4 was also detected in ALS and PD patients [255]. In addition to TLR4, also CD14 is important for the activation of inflammatory responses and phagocytosis upon exposure of microglia to β-amyloid [251, 252]. In agreement, an elevated level of CD14 was detected in brains from murine models of AD, PD, and ALS, and from AD patients [256–258]. However, the outcome of a CD14 engagement in AD can be both beneficial and detrimental [259]. The elevation of the amounts of TLR4 and CD14 in microglia and exaggerated production of pro-inflammatory mediators can result from disturbances in endo-lysosomal functioning in analogy to the LSD described above. This assumption is supported by the fact that among the features of AD, PD and ALS are defects of endo-lysosomes caused by mutations of genes encoding proteins involved directly or indirectly in their functioning [260, 261].

Recent data link TLR4 activation with the inflammatory process induced by cerebral stroke and leading to ischemic brain damage. TLR4 was upregulated after ischemia-induced stroke, and mice deficient in TLR4 exhibited less-severe cerebral infarction and neurological deficit compared with wild-type counterparts [200]. TLR4 is also associated with the development of allergies and some autoimmune diseases, like Crohn's disease, asthma, and type 1 diabetes [262–264]. Further studies are required to establish the contribution of disturbances in TLR4 trafficking to these processes.

In summary, the involvement of TLR4-triggered signaling in the development and progression of multiple diseases is often linked with its disturbed trafficking through the endo-lysosomal compartment, as demonstrated for LSD and strongly suggested for neurodegenerative diseases. It remains an open question whether defects in TLR4 trafficking contribute also to the severity of acute and chronic inflammation caused, respectively, by infection and metabolic endotoxemia, the latter known to participate in the development of type 2 diabetes. A comprehensive knowledge of the TLR4 trafficking pathways and the role of CD14 in these processes is key to understanding mechanisms regulating TLR4-induced inflammatory response in both infectious and non-infectious diseases. This in turn should facilitate the design of new therapeutic strategies for treatment of these diseases.

## Abbreviations

AD: Alzheimer's disease
ALS: Amyotrophic lateral sclerosis
AOHA: Acyloxyacyl hydrolase
Arf6: ADP-ribosylation factor 6
BMDC: Bone marrow-derived dendritic cells
BMDM: Bone marrow-derived macrophages
CF: Cystic fibrosis
CFTR: Cystic fibrosis transmembrane conductance regulator
CHS: Chédiak–Higashi syndrome
CLIC: Clathrin-independent carrier
Dab2: Disabled-2
DAMP: Damage-associated molecular pattern
DC: Dendritic cells
ERC: Endosomal recycling compartment
GAG: Glycosaminoglycan
GEEC: GPI-AP-enriched early endosomal compartment
GMFG: Glia maturation factor-γ
GPI-AP: Glycosylphosphatidylinositol-anchored proteins
HMGB1: High mobility group box protein 1
IP_3_: Inositol 1,4,5-trisphosphate
IFN: Interferon
IKK: IκB kinase
IL: Interleukin
IRAK: Interleukin-1 receptor-associated kinase
IRF: Interferon regulatory factor
LBP: LPS-binding protein
LPS: Lipopolysaccharide
LSD: Lysosomal storage disorders
MHC: Major histocompatibility complex
MPS: Mucopolysaccharidosis
MVB: Multivesicular bodies
NPC: Niemann–Pick's type C disease
OMV: Outer membrane vesicles
oxPAPC: Oxidized 1-palmitoyl-2-arachidonyl-*sn*-glycero-3- phosphorylcholine
PAMP: Pathogen-associated molecular pattern
PD: Parkinson's disease
PI: Phosphatidylinositol
PI(4,5)P_2_: Phosphatidylinositol 4,5-bisphosphate
PI(3,4,5)P_3_: Phosphatidylinositol 3,4,5-trisphosphate
PIP5KI: Phosphatidylinositol 4-phosphate 5-kinase type I
PLCγ2: Phospholipase Cγ2
PRR: Pattern recognition receptor
PS: Phosphatidylserine
RIPK1: Receptor-interacting serine/threonine-protein kinase 1
sCD14: Soluble form of CD14
TAK1: Transforming growth factor-beta-activated kinase 1
TBK1: TANK binding kinase 1
TIR: Toll/IL-1R homology domain
TLR: Toll-like receptor
TNF-α: Tumor necrosis factor-α
Vps: Vacuolar protein sorting-associated protein

## References

[1] Kumar H, Kawai T, Akira S (2011). Pathogen recognition by the innate immune system. Int Rev Immunol.

[2] Brubaker SW, Bonham KS, Zanoni I, Kagan JC (2015). Innate immune pattern recognition: a cell biological perspective. Annu Rev Immunol.

[3] Płóciennikowska A, Hromada-Judycka A, Borzęcka K, Kwiatkowska K (2015). Co-operation of TLR4 and raft proteins in LPS-induced pro-inflammatory signaling. Cell Mol Life Sci.

[4] Cao X (2016). Self-regulation and cross-regulation of pattern-recognition receptor signalling in health and disease. Nat Rev Immunol.

[5] Fitzgerald KA, Kagan JC (2020). Toll-like receptors and the control of immunity. Cell.

[6] Poltorak A, Smirnova I, He X, Liu MY, Van Huffel C, McNally O, Birdwell D, Alejos E, Silva M, Du X, Thompson P, Chan EK, Ledesma J, Roe B, Clifton S, Vogel SN, Beutler B (1998). Genetic and physical mapping of the Lps locus: identification of the toll-4 receptor as a candidate gene in the critical region. Blood Cells Mol Dis.

[7] Yang H, Wang H, Ju Z, Ragab AA, Lundbäck P, Long W, Valdes-Ferrer SI, He M, Pribis JP, Li J, Lu B, Gero D, Szabo C, Antoine DJ, Harris HE, Golenbock DT, Meng J, Roth J, Chavan SS, Andersson U, Billiar TR, Tracey KJ, Al-Abed Y (2015). MD-2 is required for disulfide HMGB1-dependent TLR4 signaling. J Exp Med.

[8] Jiang D, Liang J, Fan J, Yu S, Chen S, Luo Y, Prestwich GD, Mascarenhas MM, Garg HG, Quinn DA, Homer RJ, Goldstein DR, Bucala R, Lee PJ, Medzhitov R, Noble PW (2005). Regulation of lung injury and repair by Toll-like receptors and hyaluronan. Nat Med.

[9] Manček-Keber M, Jerala R (2015). Postulates for validating TLR4 agonists. Eur J Immunol.

[10] Kim HM, Kim Y-M (2018). HMGB1: LPS delivery vehicle for caspase-11-mediated pyroptosis. Immunity.

[11] Miyake K, Nagai Y, Akashi S, Nagafuku M, Ogata M, Kosugi A (2002). Essential role of MD-2 in B-cell responses to lipopolysaccharide and Toll-like receptor 4 distribution. J Endotoxin Res.

[12] Meng J, Gong M, Björkbacka H (1950). Golenbock DT (2011) Genome-wide expression profiling and mutagenesis studies reveal that lipopolysaccharide responsiveness appears to be absolutely dependent on TLR4 and MD-2 expression and is dependent upon intermolecular ionic interactions. J Immunol.

[13] Park BS, Song DH, Kim HM, Choi B-S, Lee H, Lee J-O (2009). The structural basis of lipopolysaccharide recognition by the TLR4-MD-2 complex. Nature.

[14] Park BS, Lee J-O (2013). Recognition of lipopolysaccharide pattern by TLR4 complexes. Exp Mol Med.

[15] Steimle A, Autenrieth IB, Frick J-S (2016). Structure and function: Lipid A modifications in commensals and pathogens. Int J Med Microbiol.

[16] Kelley SL, Lukk T, Nair SK (1950). Tapping RI (2013) The crystal structure of human soluble CD14 reveals a bent solenoid with a hydrophobic amino-terminal pocket. J Immunol.

[17] Tan Y, Zanoni I, Cullen TW, Goodman AL, Kagan JC (2015). Mechanisms of toll-like receptor 4 endocytosis reveal a common immune-evasion strategy used by pathogenic and commensal bacteria. Immunity.

[18] Cani PD, Bibiloni R, Knauf C, Waget A, Neyrinck AM, Delzenne NM, Burcelin R (2008). Changes in gut microbiota control metabolic endotoxemia-induced inflammation in high-fat diet-induced obesity and diabetes in mice. Diabetes.

[19] Poltorak A, He X, Smirnova I, Liu MY, Van Huffel C, Du X, Birdwell D, Alejos E, Silva M, Galanos C, Freudenberg M, Ricciardi-Castagnoli P, Layton B, Beutler B (1998). Defective LPS signaling in C3H/HeJ and C57BL/10ScCr mice: mutations in Tlr4 gene. Science.

[20] Mayr FB, Yende S, Angus DC (2014). Epidemiology of severe sepsis Virulence.

[21] Cani PD, Amar J, Iglesias MA, Poggi M, Knauf C, Bastelica D, Neyrinck AM, Fava F, Tuohy KM, Chabo C, Waget A, Delmée E, Cousin B, Sulpice T, Chamontin B, Ferrières J, Tanti J-F, Gibson GR, Casteilla L, Delzenne NM, Alessi MC, Burcelin R (2007). Metabolic endotoxemia initiates obesity and insulin resistance. Diabetes.

[22] Velloso LA, Folli F, Saad MJ (2015). TLR4 at the crossroads of nutrients, gut microbiota, and metabolic inflammation. Endocr Rev.

[23] Molteni M, Gemma S, Rossetti C (2016). The role of Toll-Like receptor 4 in infectious and noninfectious inflammation. Mediators Inflamm.

[24] Gambuzza M, Licata N, Palella E, Celi D, Foti Cuzzola V, Italiano D, Marino S, Bramanti P (2011). Targeting Toll-like receptors: emerging therapeutics for multiple sclerosis management. J Neuroimmunol.

[25] Li Z, Block MS, Vierkant RA, Fogarty ZC, Winham SJ, Visscher DW, Kalli KR, Wang C, Goode EL (2016). The inflammatory microenvironment in epithelial ovarian cancer: a role for TLR4 and MyD88 and related proteins. Tumour Biol.

[26] Deng M, Scott MJ, Loughran P, Gibson G, Sodhi C, Watkins S, Hackam D (1950). Billiar TR (2013) Lipopolysaccharide clearance, bacterial clearance, and systemic inflammatory responses are regulated by cell type-specific functions of TLR4 during sepsis. J Immunol.

[27] Zhang M, Zou L, Feng Y, Chen Y-J, Zhou Q, Ichinose F, Chao W (2014). Toll-like receptor 4 is essential to preserving cardiac function and survival in low-grade polymicrobial sepsis. Anesthesiology.

[28] Vaure C, Liu Y (2014). A Comparative Review of Toll-Like Receptor 4 Expression and Functionality in Different Animal Species. Front Immunol.

[29] Mahnke K, Becher E, Ricciardi-Castagnoli P, Luger TA, Schwarz T, Grabbe S (1997). CD14 is expressed by subsets of murine dendritic cells and upregulated by lipopolysaccharide. Adv Exp Med Biol.

[30] Sabroe I, Jones EC, Usher LR, Whyte MKB (1950). Dower SK (2002) Toll-like receptor (TLR)2 and TLR4 in human peripheral blood granulocytes: a critical role for monocytes in leukocyte lipopolysaccharide responses. J Immunol.

[31] Gangloff SC, Zähringer U, Blondin C, Guenounou M, Silver J (1950). Goyert SM (2005) Influence of CD14 on ligand interactions between lipopolysaccharide and its receptor complex. J Immunol.

[32] Jiang Z, Georgel P, Du X, Shamel L, Sovath S, Mudd S, Huber M, Kalis C, Keck S, Galanos C, Freudenberg M, Beutler B (2005). CD14 is required for MyD88-independent LPS signaling. Nat Immunol.

[33] Zanoni I, Ostuni R, Marek LR, Barresi S, Barbalat R, Barton GM, Granucci F, Kagan JC (2011). CD14 controls the LPS-induced endocytosis of Toll-like receptor 4. Cell.

[34] Borzęcka K, Płóciennikowska A, Björkelund H, Sobota A, Kwiatkowska K (2013). CD14 mediates binding of high doses of LPS but is dispensable for TNF-α production. Mediators Inflamm.

[35] Meissner F, Scheltema RA, Mollenkopf H-J, Mann M (2013). Direct proteomic quantification of the secretome of activated immune cells. Science.

[36] Björkbacka H, Fitzgerald KA, Huet F, Li X, Gregory JA, Lee MA, Ordija CM, Dowley NE, Golenbock DT, Freeman MW (2004). The induction of macrophage gene expression by LPS predominantly utilizes Myd88-independent signaling cascades. Physiol Genomics.

[37] Hirotani T, Yamamoto M, Kumagai Y, Uematsu S, Kawase I, Takeuchi O, Akira S (2005). Regulation of lipopolysaccharide-inducible genes by MyD88 and Toll/IL-1 domain containing adaptor inducing IFN-beta. Biochem Biophys Res Commun.

[38] Yamamoto M, Sato S, Hemmi H, Hoshino K, Kaisho T, Sanjo H, Takeuchi O, Sugiyama M, Okabe M, Takeda K, Akira S (2003). Role of adaptor TRIF in the MyD88-independent toll-like receptor signaling pathway. Science.

[39] Yamamoto M, Sato S, Hemmi H, Sanjo H, Uematsu S, Kaisho T, Hoshino K, Takeuchi O, Kobayashi M, Fujita T, Takeda K, Akira S (2002). Essential role for TIRAP in activation of the signalling cascade shared by TLR2 and TLR4. Nature.

[40] Kagan JC, Su T, Horng T, Chow A, Akira S, Medzhitov R (2008). TRAM couples endocytosis of Toll-like receptor 4 to the induction of interferon-beta. Nat Immunol.

[41] Husebye H, Halaas Ø, Stenmark H, Tunheim G, Sandanger Ø, Bogen B, Brech A, Latz E, Espevik T (2006). Endocytic pathways regulate Toll-like receptor 4 signaling and link innate and adaptive immunity. EMBO J.

[42] Yu B, Wright SD (1996). Catalytic properties of lipopolysaccharide (LPS) binding protein transfer of LPS to soluble CD14. J Biol Chem.

[43] Iovine N, Eastvold J, Elsbach P, Weiss JP, Gioannini TL (2002). The carboxyl-terminal domain of closely related endotoxin-binding proteins determines the target of protein-lipopolysaccharide complexes. J Biol Chem.

[44] Gioannini TL, Teghanemt A, Zhang D, Levis EN, Weiss JP (2005). Monomeric endotoxin:protein complexes are essential for TLR4-dependent cell activation. J Endotoxin Res.

[45] Tsukamoto H, Takeuchi S, Kubota K, Kobayashi Y, Kozakai S, Ukai I, Shichiku A, Okubo M, Numasaki M, Kanemitsu Y, Matsumoto Y, Nochi T, Watanabe K, Aso H, Tomioka Y (2018). Lipopolysaccharide (LPS)-binding protein stimulates CD14-dependent Toll-like receptor 4 internalization and LPS-induced TBK1-IKKϵ-IRF3 axis activation. J Biol Chem.

[46] Ryu J-K, Kim SJ, Rah S-H, Kang JI, Jung HE, Lee D, Lee HK, Lee J-O, Park BS, Yoon T-Y, Kim HM (2017). Reconstruction of LPS transfer cascade reveals structural determinants within LBP, CD14, and TLR4-MD2 for efficient LPS recognition and transfer. Immunity.

[47] Funda DP, Tucková L, Farré MA, Iwase T, Moro I, Tlaskalová-Hogenová H (2001). CD14 is expressed and released as soluble CD14 by human intestinal epithelial cells in vitro: lipopolysaccharide activation of epithelial cells revisited. Infect Immun.

[48] de Buhr MF, Hedrich H-J, Westendorf AM, Obermeier F, Hofmann C, Zschemisch N-H, Buer J, Bumann D, Goyert SM, Bleich A (2009). Analysis of Cd14 as a genetic modifier of experimental inflammatory bowel disease (IBD) in mice. Inflamm Bowel Dis.

[49] Fernández-Real JM, Pérez del Pulgar S, Luche E, Moreno-Navarrete JM, Waget A, Serino M, Sorianello E, Sánchez-Pla A, Pontaque FC, Vendrell J, Chacón MR, Ricart W, Burcelin R, Zorzano A (2011). CD14 Modulates Inflammation-Driven Insulin Resistance. Diabetes.

[50] Roy S, Karmakar M, Pearlman E (2014). CD14 mediates Toll-like receptor 4 (TLR4) endocytosis and spleen tyrosine kinase (Syk) and interferon regulatory transcription factor 3 (IRF3) activation in epithelial cells and impairs neutrophil infiltration and Pseudomonas aeruginosa killing in vivo. J Biol Chem.

[51] Wu Z, Zhang Z, Lei Z, Lei P (2019). CD14: Biology and role in the pathogenesis of disease. Cytokine Growth Factor Rev.

[52] Bufler P, Stiegler G, Schuchmann M, Hess S, Krüger C, Stelter F, Eckerskorn C, Schütt C, Engelmann H (1995). Soluble lipopolysaccharide receptor (CD14) is released via two different mechanisms from human monocytes and CD14 transfectants. Eur J Immunol.

[53] Delgado M, Leceta J, Abad C, Martinez C, Ganea D, Gomariz RP (1999). Shedding of membrane-bound CD14 from lipopolysaccharide-stimulated macrophages by vasoactive intestinal peptide and pituitary adenylate cyclase activating polypeptide. J Neuroimmunol.

[54] Arai Y, Mizugishi K, Nonomura K, Naitoh K, Takaori-Kondo A, Yamashita K (2015). Phagocytosis by human monocytes is required for the secretion of presepsin. J Infect Chemother.

[55] Duchow J, Marchant A, Crusiaux A, Husson C, Alonso-Vega C, De Groote D, Neve P, Goldman M (1993). Impaired phagocyte responses to lipopolysaccharide in paroxysmal nocturnal hemoglobinuria. Infect Immun.

[56] Landmann R, Reber AM, Sansano S, Zimmerli W (1996). Function of soluble CD14 in serum from patients with septic shock. J Infect Dis.

[57] da Silva CJ, Soldau K, Christen U, Tobias PS, Ulevitch RJ (2001). Lipopolysaccharide is in close proximity to each of the proteins in its membrane receptor complex. transfer from CD14 to TLR4 and MD-2. J Biol Chem.

[58] Kim J-I, Lee CJ, Jin MS, Lee C-H, Paik S-G, Lee H, Lee J-O (2005). Crystal structure of CD14 and its implications for lipopolysaccharide signaling. J Biol Chem.

[59] Hajjar AM, Ernst RK, Tsai JH, Wilson CB, Miller SI (2002). Human Toll-like receptor 4 recognizes host-specific LPS modifications. Nat Immunol.

[60] Sprong T, van der Ley P, Abdollahi-Roodsaz S, Joosten L, van der Meer J, Netea M, van Deuren M (2011). Neisseria meningitidis lipid A mutant LPSs function as LPS antagonists in humans by inhibiting TLR 4-dependent cytokine production. Innate Immun.

[61] Hajjar AM, Ernst RK, Fortuno ES, Brasfield AS, Yam CS, Newlon LA, Kollmann TR, Miller SI, Wilson CB (2012). Humanized TLR4/MD-2 mice reveal LPS recognition differentially impacts susceptibility to Yersinia pestis and Salmonella enterica. PLoS Pathog.

[62] Ohto U, Fukase K, Miyake K, Shimizu T (2012). Structural basis of species-specific endotoxin sensing by innate immune receptor TLR4/MD-2. Proc Natl Acad Sci U S A.

[63] Meng J, Lien E, Golenbock DT (2010). MD-2-mediated ionic interactions between lipid A and TLR4 are essential for receptor activation. J Biol Chem.

[64] Zhang Y, Gaekwad J, Wolfert MA, Boons G-J (2007). Modulation of innate immune responses with synthetic lipid A derivatives. J Am Chem Soc.

[65] Pizzuto M, Lonez C, Baroja-Mazo A, Martínez-Banaclocha H, Tourlomousis P, Gangloff M, Pelegrin P, Ruysschaert J-M, Gay NJ, Bryant CE (2019). Saturation of acyl chains converts cardiolipin from an antagonist to an activator of Toll-like receptor-4. Cell Mol Life Sci.

[66] Anwar MA, Panneerselvam S, Shah M, Choi S (2015). Insights into the species-specific TLR4 signaling mechanism in response to Rhodobacter sphaeroides lipid A detection. Sci Rep.

[67] Mullarkey M, Rose JR, Bristol J, Kawata T, Kimura A, Kobayashi S, Przetak M, Chow J, Gusovsky F, Christ WJ, Rossignol DP (2003). Inhibition of endotoxin response by e5564, a novel Toll-like receptor 4-directed endotoxin antagonist. J Pharmacol Exp Ther.

[68] Kim HM, Park BS, Kim J-I, Kim SE, Lee J, Oh SC, Enkhbayar P, Matsushima N, Lee H, Yoo OJ, Lee J-O (2007). Crystal structure of the TLR4-MD-2 complex with bound endotoxin antagonist Eritoran. Cell.

[69] Panter G, Jerala R (2011). The ectodomain of the Toll-like receptor 4 prevents constitutive receptor activation. J Biol Chem.

[70] Fitzgerald KA, Palsson-McDermott EM, Bowie AG, Jefferies CA, Mansell AS, Brady G, Brint E, Dunne A, Gray P, Harte MT, McMurray D, Smith DE, Sims JE, Bird TA, O’Neill LA (2001). Mal (MyD88-adapter-like) is required for Toll-like receptor-4 signal transduction. Nature.

[71] Horng T, Barton GM, Flavell RA, Medzhitov R (2002). The adaptor molecule TIRAP provides signalling specificity for Toll-like receptors. Nature.

[72] Bonham KS, Orzalli MH, Hayashi K, Wolf AI, Glanemann C, Weninger W, Iwasaki A, Knipe DM, Kagan JC (2014). A promiscuous lipid-binding protein diversifies the subcellular sites of toll-like receptor signal transduction. Cell.

[73] Kagan JC, Medzhitov R (2006). Phosphoinositide-mediated adaptor recruitment controls Toll-like receptor signaling. Cell.

[74] Aksoy E, Taboubi S, Torres D, Delbauve S, Hachani A, Whitehead MA, Pearce WP, Berenjeno IM, Nock G, Filloux A, Beyaert R, Flamand V, Vanhaesebroeck B (2012). The p110δ isoform of the kinase PI(3)K controls the subcellular compartmentalization of TLR4 signaling and protects from endotoxic shock. Nat Immunol.

[75] Motshwene PG, Moncrieffe MC, Grossmann JG, Kao C, Ayaluru M, Sandercock AM, Robinson CV, Latz E, Gay NJ (2009). An oligomeric signaling platform formed by the Toll-like receptor signal transducers MyD88 and IRAK-4. J Biol Chem.

[76] Lin S-C, Lo Y-C, Wu H (2010). Helical assembly in the MyD88-IRAK4-IRAK2 complex in TLR/IL-1R signalling. Nature.

[77] Lu Y-C, Yeh W-C, Ohashi PS (2008). LPS/TLR4 signal transduction pathway. Cytokine.

[78] Laird MHW, Rhee SH, Perkins DJ, Medvedev AE, Piao W, Fenton MJ, Vogel SN (2009). TLR4/MyD88/PI3K interactions regulate TLR4 signaling. J Leukoc Biol.

[79] Kawai T, Akira S (2011). Toll-like receptors and their crosstalk with other innate receptors in infection and immunity. Immunity.

[80] Kawai T, Takeuchi O, Fujita T, Inoue J, Mühlradt PF, Sato S, Hoshino K (1950). Akira S (2001) Lipopolysaccharide stimulates the MyD88-independent pathway and results in activation of IFN-regulatory factor 3 and the expression of a subset of lipopolysaccharide-inducible genes. J Immunol.

[81] Odendall C, Voak AA (1950). Kagan JC (2017) Type III interferons are commonly induced by bacteria-sensing TLRs, and reinforce epithelial barriers during infection. J Immunol.

[82] Chanteux H, Guisset AC, Pilette C, Sibille Y (2007). LPS induces IL-10 production by human alveolar macrophages via MAPKinases- and Sp1-dependent mechanisms. Respir Res.

[83] Troutman TD, Hu W, Fulenchek S, Yamazaki T, Kurosaki T, Bazan JF, Pasare C (2012). Role for B-cell adapter for PI3K (BCAP) as a signaling adapter linking Toll-like receptors (TLRs) to serine/threonine kinases PI3K/Akt. Proc Natl Acad Sci.

[84] Ni M, MacFarlane AW, Toft M, Lowell CA, Campbell KS, Hamerman JA (2012). B-cell adaptor for PI3K (BCAP) negatively regulates Toll-like receptor signaling through activation of PI3K. Proc Natl Acad Sci.

[85] Borzęcka-Solarz K, Dembińska J, Hromada-Judycka A, Traczyk G, Ciesielska A, Ziemlińska E, Świątkowska A, Kwiatkowska K (2017). Association of Lyn kinase with membrane rafts determines its negative influence on LPS-induced signaling. Mol Biol Cell.

[86] Perkins DJ, Richard K, Hansen A-M, Lai W, Nallar S, Koller B, Vogel SN (2018). Autocrine-paracrine prostaglandin E2 signaling restricts TLR4 internalization and TRIF signaling. Nat Immunol.

[87] Everts B, Amiel E, Huang SC-C, Smith AM, Chang C-H, Lam WY, Redmann V, Freitas TC, Blagih J, van der Windt GJW, Artyomov MN, Jones RG, Pearce EL, Pearce EJ (2014). TLR-driven early glycolytic reprogramming via the kinases TBK1-IKKɛ supports the anabolic demands of dendritic cell activation. Nat Immunol.

[88] Tan Y, Kagan JC (2019). Innate Immune signaling organelles display natural and programmable signaling flexibility. Cell.

[89] Langston PK, Nambu A, Jung J, Shibata M, Aksoylar HI, Lei J, Xu P, Doan MT, Jiang H, MacArthur MR, Gao X, Kong Y, Chouchani ET, Locasale JW, Snyder NW, Horng T (2019). Glycerol phosphate shuttle enzyme GPD2 regulates macrophage inflammatory responses. Nat Immunol.

[90] Marongiu L, Gornati L, Artuso I, Zanoni I, Granucci F (2019). Below the surface: The inner lives of TLR4 and TLR9. J Leukoc Biol.

[91] Oshiumi H, Sasai M, Shida K, Fujita T, Matsumoto M, Seya T (2003). TIR-containing adapter molecule (TICAM)-2, a bridging adapter recruiting to toll-like receptor 4 TICAM-1 that induces interferon-beta. J Biol Chem.

[92] Latz E, Visintin A, Lien E, Fitzgerald KA, Monks BG, Kurt-Jones EA, Golenbock DT, Espevik T (2002). Lipopolysaccharide rapidly traffics to and from the Golgi apparatus with the toll-like receptor 4-MD-2-CD14 complex in a process that is distinct from the initiation of signal transduction. J Biol Chem.

[93] Rowe DC, McGettrick AF, Latz E, Monks BG, Gay NJ, Yamamoto M, Akira S, O’Neill LA, Fitzgerald KA, Golenbock DT (2006). The myristoylation of TRIF-related adaptor molecule is essential for Toll-like receptor 4 signal transduction. Proc Natl Acad Sci U S A.

[94] Tanimura N, Saitoh S, Matsumoto F, Akashi-Takamura S, Miyake K (2008). Roles for LPS-dependent interaction and relocation of TLR4 and TRAM in TRIF-signaling. Biochem Biophys Res Commun.

[95] Husebye H, Aune MH, Stenvik J, Samstad E, Skjeldal F, Halaas O, Nilsen NJ, Stenmark H, Latz E, Lien E, Mollnes TE, Bakke O, Espevik T (2010). The Rab11a GTPase controls Toll-like receptor 4-induced activation of interferon regulatory factor-3 on phagosomes. Immunity.

[96] Liu S, Cai X, Wu J , Cong Q, Chen X, Li T, Du F, Ren J, Wu YT, Grishin NV, Chen ZJ (2015) Phosphorylation of innate immune adaptor proteins MAVS, STING, and TRIF induces IRF3 activation | Science. https://science.sciencemag.org/content/347/6227/aaa2630 10.1126/science.aaa2630 25636800

[97] Häcker H, Redecke V, Blagoev B, Kratchmarova I, Hsu L-C, Wang GG, Kamps MP, Raz E, Wagner H, Häcker G, Mann M, Karin M (2006). Specificity in Toll-like receptor signalling through distinct effector functions of TRAF3 and TRAF6. Nature.

[98] Chang EY, Guo B, Doyle SE, Cheng G (2007). Cutting edge: involvement of the type I IFN production and signaling pathway in lipopolysaccharide-induced IL-10 production. J Immunol.

[99] Sato S, Sugiyama M, Yamamoto M, Watanabe Y, Kawai T, Takeda K, Akira S (2003). Toll/IL-1 receptor domain-containing adaptor inducing IFN-β (TRIF) associates with TNF receptor-associated factor 6 and TANK-binding kinase 1, and activates two distinct transcription factors, NF-κB and IFN-regulatory factor-3, in the Toll-like receptor signaling. J Immunol.

[100] Cusson-Hermance N, Khurana S, Lee TH, Fitzgerald KA, Kelliher MA (2005). Rip1 mediates the Trif-dependent Toll-like receptor 3- and 4-induced NF-κB activation but does not contribute to interferon regulatory factor 3 activation. J Biol Chem.

[101] Kaiser WJ (1950). Offermann MK (2005) Apoptosis induced by the toll-like receptor adaptor TRIF is dependent on its receptor interacting protein homotypic interaction motif. J Immunol.

[102] He S, Liang Y, Shao F, Wang X (2011). Toll-like receptors activate programmed necrosis in macrophages through a receptor-interacting kinase-3-mediated pathway. Proc Natl Acad Sci.

[103] Kaiser WJ, Sridharan H, Huang C, Mandal P, Upton JW, Gough PJ, Sehon CA, Marquis RW, Bertin J, Mocarski ES (2013). Toll-like receptor 3-mediated necrosis via TRIF, RIP3, and MLKL. J Biol Chem.

[104] Najjar M, Saleh D, Zelic M, Nogusa S, Shah S, Tai A, Finger JN, Polykratis A, Gough PJ, Bertin J, Whalen MJ, Pasparakis M, Balachandran S, Kelliher M, Poltorak A, Degterev A (2016). RIPK1 and RIPK3 kinases promote cell-death-independent inflammation by Toll-like receptor 4. Immunity.

[105] Yi Y-S (2017). Caspase-11 non-canonical inflammasome: a critical sensor of intracellular lipopolysaccharide in macrophage-mediated inflammatory responses. Immunology.

[106] Barker JH, Weiss JP (2019). Detecting lipopolysaccharide in the cytosol of mammalian cells: Lessons from MD-2/TLR4. J Leukoc Biol.

[107] Kelley N, Jeltema D, Duan Y, He Y (2019). The NLRP3 inflammasome: an overview of mechanisms of activation and regulation. Int J Mol Sci.

[108] Swanson KV, Deng M, Ting JP-Y (2019). The NLRP3 inflammasome: molecular activation and regulation to therapeutics. Nat Rev Immunol.

[109] Evavold CL, Ruan J, Tan Y, Xia S, Wu H, Kagan JC (2018). The pore-forming protein gasdermin D regulates interleukin-1 secretion from living macrophages. Immunity.

[110] Franchi L, Eigenbrod T (1950). Núñez G (2009) Cutting edge: TNF-alpha mediates sensitization to ATP and silica via the NLRP3 inflammasome in the absence of microbial stimulation. J Immunol.

[111] Bauerfeld CP, Rastogi R, Pirockinaite G, Lee I, Hüttemann M, Monks B, Birnbaum MJ, Franchi L, Nuñez G (1950). Samavati L (2012) TLR4-mediated AKT activation is MyD88/TRIF dependent and critical for induction of oxidative phosphorylation and mitochondrial transcription factor A in murine macrophages. J Immunol.

[112] Vanaja SK, Russo AJ, Behl B, Banerjee I, Yankova M, Deshmukh SD, Rathinam VAK (2016). Bacterial outer membrane vesicles mediate cytosolic localization of LPS and caspase-11 activation. Cell.

[113] Deng M, Tang Y, Li W, Wang X, Zhang R, Zhang X, Zhao X, Liu J, Tang C, Liu Z, Huang Y, Peng H, Xiao L, Tang D, Scott MJ, Wang Q, Liu J, Xiao X, Watkins S, Li J, Yang H, Wang H, Chen F, Tracey KJ, Billiar TR, Lu B (2018). The endotoxin delivery protein HMGB1 mediates caspase-11-dependent lethality in sepsis. Immunity.

[114] Netea MG, Nold-Petry CA, Nold MF, Joosten LAB, Opitz B, van der Meer JHM, van de Veerdonk FL, Ferwerda G, Heinhuis B, Devesa I, Funk CJ, Mason RJ, Kullberg BJ, Rubartelli A, van der Meer JWM, Dinarello CA (2009). Differential requirement for the activation of the inflammasome for processing and release of IL-1beta in monocytes and macrophages. Blood.

[115] Gaidt MM, Ebert TS, Chauhan D, Schmidt T, Schmid-Burgk JL, Rapino F, Robertson AAB, Cooper MA, Graf T, Hornung V (2016). Human monocytes engage an alternative inflammasome pathway. Immunity.

[116] Zewinger S, Reiser J, Jankowski V, Alansary D, Hahm E, Triem S, Klug M, Schunk SJ, Schmit D, Kramann R, Körbel C, Ampofo E, Laschke MW, Selejan S-R, Paschen A, Herter T, Schuster S, Silbernagel G, Sester M, Sester U, Aßmann G, Bals R, Kostner G, Jahnen-Dechent W, Menger MD, Rohrer L, März W, Böhm M, Jankowski J, Kopf M, Latz E, Niemeyer BA, Fliser D, Laufs U, Speer T (2020). Apolipoprotein C3 induces inflammation and organ damage by alternative inflammasome activation. Nat Immunol.

[117] Pasare C, Medzhitov R (2005). Control of B-cell responses by Toll-like receptors. Nature.

[118] Kaisho T, Takeuchi O, Kawai T, Hoshino K (1950). Akira S (2001) Endotoxin-induced maturation of MyD88-deficient dendritic cells. J Immunol.

[119] Hoebe K, Janssen EM, Kim SO, Alexopoulou L, Flavell RA, Han J, Beutler B (2003). Upregulation of costimulatory molecules induced by lipopolysaccharide and double-stranded RNA occurs by Trif-dependent and Trif-independent pathways. Nat Immunol.

[120] Shen H, Tesar BM, Walker WE (1950). Goldstein DR (2008) dual signaling of MyD88 and TRIF are critical for maximal TLR4-induced dendritic cell maturation. J Immunol.

[121] Turley SJ, Inaba K, Garrett WS, Ebersold M, Unternaehrer J, Steinman RM, Mellman I (2000). Transport of peptide-MHC class II complexes in developing dendritic cells. Science.

[122] Trombetta ES, Ebersold M, Garrett W, Pypaert M, Mellman I (2003). Activation of lysosomal function during dendritic cell maturation. Science.

[123] Nair-Gupta P, Baccarini A, Tung N, Seyffer F, Florey O, Huang Y, Banerjee M, Overholtzer M, Roche PA, Tampé R, Brown BD, Amsen D, Whiteheart SW, Blander JM (2014). TLR signals induce phagosomal MHC-I delivery from the endosomal recycling compartment to allow cross-presentation. Cell.

[124] Zanoni I, Ostuni R, Capuano G, Collini M, Caccia M, Ronchi AE, Rocchetti M, Mingozzi F, Foti M, Chirico G, Costa B, Zaza A, Ricciardi-Castagnoli P, Granucci F (2009). CD14 regulates the dendritic cell life cycle after LPS exposure through NFAT activation. Nature.

[125] Ling GS, Bennett J, Woollard KJ, Szajna M, Fossati-Jimack L, Taylor PR, Scott D, Franzoso G, Cook HT, Botto M (2014). Integrin CD11b positively regulates TLR4-induced signalling pathways in dendritic cells but not in macrophages. Nat Commun.

[126] Terstappen LW, Hollander Z, Meiners H, Loken MR (1990). Quantitative comparison of myeloid antigens on five lineages of mature peripheral blood cells. J Leukoc Biol.

[127] Varadaradjalou S, Féger F, Thieblemont N, Hamouda NB, Pleau J-M, Dy M, Arock M (2003). Toll-like receptor 2 (TLR2) and TLR4 differentially activate human mast cells. Eur J Immunol.

[128] Brenner SA, Zacheja S, Schäffer M, Feilhauer K, Bischoff SC, Lorentz A (2014). Soluble CD14 is essential for lipopolysaccharide-dependent activation of human intestinal mast cells from macroscopically normal as well as Crohn’s disease tissue. Immunology.

[129] Reynolds JM, Martinez GJ, Chung Y, Dong C (2012). Toll-like receptor 4 signaling in T cells promotes autoimmune inflammation. Proc Natl Acad Sci.

[130] Keck S, Müller I, Fejer G, Savic I, Tchaptchet S, Nielsen PJ, Galanos C, Huber M (1950). Freudenberg MA (2011) Absence of TRIF signaling in lipopolysaccharide-stimulated murine mast cells. J Immunol.

[131] Ikeda T, Funaba M (2003). Altered function of murine mast cells in response to lipopolysaccharide and peptidoglycan. Immunol Lett.

[132] Dietrich N, Rohde M, Geffers R, Kröger A, Hauser H, Weiss S, Gekara NO (2010). Mast cells elicit proinflammatory but not type I interferon responses upon activation of TLRs by bacteria. Proc Natl Acad Sci.

[133] Tamassia N, Moigne VL, Calzetti F, Donini M, Gasperini S, Ear T, Cloutier A, Martinez FO, Fabbri M, Locati M, Mantovani A, McDonald PP, Cassatella MA (2007). The MYD88-independent pathway is not mobilized in human neutrophils stimulated via TLR4. J Immunol.

[134] Mitroulis I, Kourtzelis I, Kambas K, Rafail S, Chrysanthopoulou A, Speletas M, Ritis K (2010). Regulation of the autophagic machinery in human neutrophils. Eur J Immunol.

[135] El-Benna J, Hurtado-Nedelec M, Marzaioli V, Marie J-C, Gougerot-Pocidalo M-A, Dang PM-C (2016). Priming of the neutrophil respiratory burst: role in host defense and inflammation. Immunol Rev.

[136] Chen S, Deng Y, He Q, Chen Y, Wang D, Sun W, He Y, Zou Z, Liang Z, Chen R, Yao L, Tao A (2020). Toll-like receptor 4 deficiency aggravates airway hyperresponsiveness and inflammation by impairing neutrophil apoptosis in a toluene diisocyanate-induced murine asthma model. Allergy Asthma Immunol Res.

[137] Smuda C, Wechsler JB, Bryce PJ (2011). TLR-induced activation of neutrophils promotes histamine production via a PI3 kinase dependent mechanism. Immunol Lett.

[138] Brinkmann V, Reichard U, Goosmann C, Fauler B, Uhlemann Y, Weiss DS, Weinrauch Y, Zychlinsky A (2004). Neutrophil extracellular traps kill bacteria. Science.

[139] Pieterse E, Rother N, Yanginlar C, Hilbrands LB, van der Vlag J (2016). Neutrophils discriminate between lipopolysaccharides of different bacterial sources and selectively release neutrophil extracellular traps. Front Immunol.

[140] Khan MA, Farahvash A, Douda DN, Licht J-C, Grasemann H, Sweezey N, Palaniyar N (2017). JNK activation turns on LPS- and Gram-negative bacteria-induced NADPH oxidase-dependent suicidal NETosis. Sci Rep.

[141] Rajaiah R, Perkins DJ, Ireland DDC, Vogel SN (2015). CD14 dependence of TLR4 endocytosis and TRIF signaling displays ligand specificity and is dissociable in endotoxin tolerance. Proc Natl Acad Sci.

[142] Iijima J, Kobayashi S, Kitazume S, Kizuka Y, Fujinawa R, Korekane H, Shibata T, Saitoh S-I, Akashi-Takamura S, Miyake K, Miyoshi E, Taniguchi N (2017). Core fucose is critical for CD14-dependent Toll-like receptor 4 signaling. Glycobiology.

[143] Nakayama K, Wakamatsu K, Fujii H, Shinzaki S, Takamatsu S, Kitazume S, Kamada Y, Takehara T, Taniguchi N, Miyoshi E (2019). Core fucose is essential glycosylation for CD14-dependent Toll-like receptor 4 and Toll-like receptor 2 signalling in macrophages. J Biochem (Tokyo).

[144] Zanoni I, Tan Y, Di Gioia M, Springstead JR, Kagan JC (2017). By capturing inflammatory lipids released from dying cells, the receptor CD14 induces inflammasome-dependent phagocyte hyperactivation. Immunity.

[145] Płóciennikowska A, Zdioruk MI, Traczyk G, Świątkowska A, Kwiatkowska K (2015). LPS-induced clustering of CD14 triggers generation of PI(4,5)P2. J Cell Sci.

[146] Płóciennikowska A, Hromada-Judycka A, Dembińska J, Roszczenko P, Ciesielska A, Kwiatkowska K (2016). Contribution of CD14 and TLR4 to changes of the PI(4,5)P2 level in LPS-stimulated cells. J Leukoc Biol.

[147] Voss OH, Murakami Y, Pena MY, Lee H-N, Tian L, Margulies DH, Street JM, Yuen PST, Qi C-F, Krzewski K, Coligan JE (2016). Lipopolysaccharide-induced CD300b receptor binding to Toll-like receptor 4 alters signaling to drive cytokine responses that enhance septic shock. Immunity.

[148] Chiang C-Y, Veckman V, Limmer K, David M (2012). Phospholipase Cγ-2 and intracellular calcium are required for lipopolysaccharide-induced Toll-like receptor 4 (TLR4) endocytosis and interferon regulatory factor 3 (IRF3) activation. J Biol Chem.

[149] Schappe MS, Szteyn K, Stremska ME, Mendu SK, Downs TK, Seegren PV, Mahoney MA, Dixit S, Krupa JK, Stipes EJ, Rogers JS, Adamson SE, Leitinger N, Desai BN (2018). Chanzyme TRPM7 mediates the Ca2+ influx essential for lipopolysaccharide-induced Toll-like receptor 4 endocytosis and macrophage activation. Immunity.

[150] Yin H, Zhou H, Kang Y, Zhang X, Duan X, Alnabhan R, Liang S, Scott DA, Lamont RJ, Shang J, Wang H (2016). Syk negatively regulates TLR4-mediated IFNβ and IL-10 production and promotes inflammatory responses in dendritic cells. Biochim Biophys Acta.

[151] Park JG, Son Y-J, Yoo BC, Yang WS, Kim JH, Kim J-H, Cho JY (2017). Syk plays a critical role in the expression and activation of IRAK1 in LPS-treated macrophages. Mediators Inflamm.

[152] Murase M, Kawasaki T, Hakozaki R, Sueyoshi T, Putri DDP, Kitai Y, Sato S, Ikawa M (1950). Kawai T (2018) Intravesicular acidification regulates lipopolysaccharide inflammation and tolerance through TLR4 trafficking. J Immunol.

[153] Van Acker T, Eyckerman S, Vande Walle L, Gerlo S, Goethals M, Lamkanfi M, Bovijn C, Tavernier J, Peelman F (2014). The small GTPase Arf6 is essential for the Tram/Trif pathway in TLR4 signaling. J Biol Chem.

[154] Ghosh M, Subramani J, Rahman MM (1950). Shapiro LH (2015) CD13 restricts TLR4 endocytic signal transduction in inflammation. J Immunol.

[155] Wang Y, Yang Y, Liu X, Wang N, Cao H, Lu Y, Zhou H, Zheng J (2012). Inhibition of clathrin/dynamin-dependent internalization interferes with LPS-mediated TRAM-TRIF-dependent signaling pathway. Cell Immunol.

[156] Ali F, Hossain MS, Sejimo S, Akashi K (2019). Plasmalogens inhibit endocytosis of Toll-like receptor 4 to attenuate the inflammatory signal in microglial cells. Mol Neurobiol.

[157] Li L, Wan T, Wan M, Liu B, Cheng R, Zhang R (2015). The effect of the size of fluorescent dextran on its endocytic pathway. Cell Biol Int.

[158] Thottacherry JJ, Sathe M, Prabhakara C, Mayor S (2019). Spoiled for choice: diverse endocytic pathways function at the cell surface. Annu Rev Cell Dev Biol.

[159] Pascual-Lucas M, Fernandez-Lizarbe S, Montesinos J, Guerri C (2014). LPS or ethanol triggers clathrin- and rafts/caveolae-dependent endocytosis of TLR4 in cortical astrocytes. J Neurochem.

[160] Czerkies M, Borzęcka K, Zdioruk MI, Płóciennikowska A, Sobota A, Kwiatkowska K (2013). An interplay between scavenger receptor A and CD14 during activation of J774 cells by high concentrations of LPS. Immunobiology.

[161] Józefowski S, Śróttek M (2017). Lipid raft-dependent endocytosis negatively regulates responsiveness of J774 macrophage-like cells to LPS by down regulating the cell surface expression of LPS receptors. Cell Immunol.

[162] Kitchens RL, Wang P (1950). Munford RS (1998) Bacterial lipopolysaccharide can enter monocytes via two CD14-dependent pathways. J Immunol.

[163] Poussin C, Foti M, Carpentier JL, Pugin J (1998). CD14-dependent endotoxin internalization via a macropinocytic pathway. J Biol Chem.

[164] Klein DCG, Skjesol A, Kers-Rebel ED, Sherstova T, Sporsheim B, Egeberg KW, Stokke BT, Espevik T, Husebye H (2015). CD14, TLR4 and TRAM Show different trafficking dynamics during LPS stimulation. Traffic.

[165] Tatematsu M, Yoshida R, Morioka Y, Ishii N, Funami K, Watanabe A, Saeki K, Seya T (1950). Matsumoto M (2016) Raftlin controls lipopolysaccharide-induced TLR4 internalization and TICAM-1 signaling in a cell type-specific manner. J Immunol.

[166] Hung W-S, Ling P, Cheng J-C, Chang S-S, Tseng C-P (2016). Disabled-2 is a negative immune regulator of lipopolysaccharide-stimulated Toll-like receptor 4 internalization and signaling. Sci Rep.

[167] Edwards AD, Diebold SS, Slack EMC, Tomizawa H, Hemmi H, Kaisho T, Akira S, Reis e Sousa C, (2003). Toll-like receptor expression in murine DC subsets: lack of TLR7 expression by CD8 alpha+ DC correlates with unresponsiveness to imidazoquinolines. Eur J Immunol.

[168] Jongbloed SL, Kassianos AJ, McDonald KJ, Clark GJ, Ju X, Angel CE, Chen C-JJ, Dunbar PR, Wadley RB, Jeet V, Vulink AJE, Hart DNJ, Radford KJ (2010). Human CD141+ (BDCA-3)+ dendritic cells (DCs) represent a unique myeloid DC subset that cross-presents necrotic cell antigens. J Exp Med.

[169] Lewis KL, Caton ML, Bogunovic M, Greter M, Grajkowska LT, Ng D, Klinakis A, Charo IF, Jung S, Gommerman JL, Ivanov II, Liu K, Merad M, Reizis B (2011). Notch2 receptor signaling controls functional differentiation of dendritic cells in the spleen and intestine. Immunity.

[170] Hémont C, Neel A, Heslan M, Braudeau C, Josien R (2013). Human blood mDC subsets exhibit distinct TLR repertoire and responsiveness. J Leukoc Biol.

[171] Villani A-C, Satija R, Reynolds G, Sarkizova S, Shekhar K, Fletcher J, Griesbeck M, Butler A, Zheng S, Lazo S, Jardine L, Dixon D, Stephenson E, Nilsson E, Grundberg I, McDonald D, Filby A, Li W, De Jager PL, Rozenblatt-Rosen O, Lane AA, Haniffa M, Regev A, Hacohen N (2017). Single-cell RNA-seq reveals new types of human blood dendritic cells, monocytes and progenitors. Science.

[172] Yin X, Yu H, Jin X, Li J, Guo H, Shi Q, Yin Z, Xu Y, Wang X, Liu R, Wang S (1950). Zhang L (2017) Human blood CD1c+ dendritic cells encompass CD5high and CD5low subsets that differ significantly in phenotype, gene expression, and functions. J Immunol.

[173] Uronen-Hansson H, Allen J, Osman M, Squires G, Klein N, Callard RE (2004). Toll-like receptor 2 (TLR2) and TLR4 are present inside human dendritic cells, associated with microtubules and the Golgi apparatus but are not detectable on the cell surface: integrity of microtubules is required for interleukin-12 production in response to internalized bacteria. Immunology.

[174] Pérez-Rodríguez MJ, Ibarra-Sánchez A, Román-Figueroa A, Pérez-Severiano F, González-Espinosa C (2020). Mutant huntingtin affects toll-like receptor 4 intracellular trafficking and cytokine production in mast cells. J Neuroinflammation.

[175] Takahashi K, Shibata T, Akashi-Takamura S, Kiyokawa T, Wakabayashi Y, Tanimura N, Kobayashi T, Matsumoto F, Fukui R, Kouro T, Nagai Y, Takatsu K, Saitoh S, Miyake K (2007). A protein associated with Toll-like receptor (TLR) 4 (PRAT4A) is required for TLR-dependent immune responses. J Exp Med.

[176] Yang Y, Liu B, Dai J, Srivastava PK, Zammit DJ, Lefrançois L, Li Z (2007). Heat shock protein gp96 is a master chaperone for Toll-like receptors and is important in the innate function of macrophages. Immunity.

[177] Visintin A, Halmen KA, Khan N, Monks BG, Golenbock DT, Lien E (2006). MD-2 expression is not required for cell surface targeting of Toll-like receptor 4 (TLR4). J Leukoc Biol.

[178] Tsukamoto H, Ihara H, Ito R, Ukai I, Suzuki N, Kimoto M, Tomioka Y, Ikeda Y (2013). MD-2-dependent human Toll-like receptor 4 monoclonal antibodies detect extracellular association of Toll-like receptor 4 with extrinsic soluble MD-2 on the cell surface. Biochem Biophys Res Commun.

[179] Wang D, Lou J, Ouyang C, Chen W, Liu Y, Liu X, Cao X, Wang J, Lu L (2010). Ras-related protein Rab10 facilitates TLR4 signaling by promoting replenishment of TLR4 onto the plasma membrane. Proc Natl Acad Sci.

[180] Akbar MA, Mandraju R, Tracy C, Hu W, Pasare C, Krämer H (2016). ARC syndrome-linked Vps33B protein is required for inflammatory endosomal maturation and signal termination. Immunity.

[181] Palsson-McDermott EM, Doyle SL, McGettrick AF, Hardy M, Husebye H, Banahan K, Gong M, Golenbock D, Espevik T, O’Neill LAJ (2009). TAG, a splice variant of the adaptor TRAM, negatively regulates the adaptor MyD88-independent TLR4 pathway. Nat Immunol.

[182] Doyle SL, Husebye H, Connolly DJ, Espevik T, O’Neill LAJ, McGettrick AF (2012). The GOLD domain-containing protein TMED7 inhibits TLR4 signalling from the endosome upon LPS stimulation. Nat Commun.

[183] Westphal A, Cheng W, Yu J, Grassl G, Krautkrämer M, Holst O, Föger N, Lee K-H (2017). Lysosomal trafficking regulator Lyst links membrane trafficking to toll-like receptor-mediated inflammatory responses. J Exp Med.

[184] Wang Y, Chen T, Han C, He D, Liu H, An H, Cai Z, Cao X (2007). Lysosome-associated small Rab GTPase Rab7b negatively regulates TLR4 signaling in macrophages by promoting lysosomal degradation of TLR4. Blood.

[185] Kinoshita D, Sakurai C, Morita M, Tsunematsu M, Hori N, Hatsuzawa K (2019). Syntaxin 11 regulates the stimulus-dependent transport of Toll-like receptor 4 to the plasma membrane by cooperating with SNAP-23 in macrophages. Mol Biol Cell.

[186] Granucci F (2018). The family of LPS signal transducers increases: the arrival of chanzymes. Immunity.

[187] Skjesol A, Yurchenko M, Bösl K, Gravastrand C, Nilsen KE, Grøvdal LM, Agliano F, Patane F, Lentini G, Kim H, Teti G, Sharma AK, Kandasamy RK, Sporsheim B, Starheim KK, Golenbock DT, Stenmark H, McCaffrey M, Espevik T, Husebye H (2019). The TLR4 adaptor TRAM controls the phagocytosis of Gram-negative bacteria by interacting with the Rab11-family interacting protein 2. PLOS Pathog.

[188] Kobayashi M, Saitoh S, Tanimura N, Takahashi K, Kawasaki K, Nishijima M, Fujimoto Y, Fukase K, Akashi-Takamura S (1950). Miyake K (2006) Regulatory roles for MD-2 and TLR4 in ligand-induced receptor clustering. J Immunol.

[189] Saitoh S-I (2009). Chaperones and transport proteins regulate TLR4 trafficking and activation. Immunobiology.

[190] Pelka K, Shibata T, Miyake K, Latz E (2016). Nucleic acid-sensing TLRs and autoimmunity: novel insights from structural and cell biology. Immunol Rev.

[191] Gangloff M (2012). Different dimerisation mode for TLR4 upon endosomal acidification?. Trends Biochem Sci.

[192] Sepulveda FE, Burgess A, Heiligenstein X, Goudin N, Ménager MM, Romao M, Côte M, Mahlaoui N, Fischer A, Raposo G, Ménasché G, de Saint BG (2015). LYST controls the biogenesis of the endosomal compartment required for secretory lysosome function. Traffic.

[193] Cullinane AR, Schäffer AA, Huizing M (2013). The BEACH is hot: a LYST of emerging roles for BEACH-domain containing proteins in human disease. Traffic.

[194] Banushi B, Forneris F, Straatman-Iwanowska A, Strange A, Lyne A-M, Rogerson C, Burden JJ, Heywood WE, Hanley J, Doykov I, Straatman KR, Smith H, Bem D, Kriston-Vizi J, Ariceta G, Risteli M, Wang C, Ardill RE, Zaniew M, Latka-Grot J, Waddington SN, Howe SJ, Ferraro F, Gjinovci A, Lawrence S, Marsh M, Girolami M, Bozec L, Mills K, Gissen P (2016). Regulation of post-Golgi LH3 trafficking is essential for collagen homeostasis. Nat Commun.

[195] Hunter MR, Hesketh GG, Benedyk TH, Gingras A-C, Graham SC (2018). Proteomic and biochemical comparison of the cellular interaction partners of human VPS33A and VPS33B. J Mol Biol.

[196] Aerbajinai W, Lee K, Chin K (1950). Rodgers GP (2013) Glia maturation factor-γ negatively modulates TLR4 signaling by facilitating TLR4 endocytic trafficking in macrophages. J Immunol.

[197] Yang M, Chen T, Han C, Li N, Wan T, Cao X (2004). Rab7b, a novel lysosome-associated small GTPase, is involved in monocytic differentiation of human acute promyelocytic leukemia cells. Biochem Biophys Res Commun.

[198] Progida C, Cogli L, Piro F, De Luca A, Bakke O, Bucci C (2010). Rab7b controls trafficking from endosomes to the TGN. J Cell Sci.

[199] Klaver EJ, van der Pouw KTCTM, Laan LC, Kringel H, Cummings RD, Bouma G, Kraal G, van Die I (2015). Trichuris suis soluble products induce Rab7b expression and limit TLR4 responses in human dendritic cells. Genes Immun.

[200] Qi J, Rong Y, Wang L, Xu J, Zhao K (2019). Rab7b overexpression-ameliorated ischemic brain damage following tMCAO Involves suppression of TLR4 and NF-κB p65. J Mol Neurosci MN.

[201] Rojas R, van Vlijmen T, Mardones GA, Prabhu Y, Rojas AL, Mohammed S, Heck AJR, Raposo G, van der Sluijs P, Bonifacino JS (2008). Regulation of retromer recruitment to endosomes by sequential action of Rab5 and Rab7. J Cell Biol.

[202] Yin J, Liu X, He Q, Zhou L, Yuan Z, Zhao S (2016). Vps35-dependent recycling of Trem2 regulates microglial function. Traffic.

[203] Ciesielska A, Sas-Nowosielska H, Kwiatkowska K (2017). Bis(monoacylglycero)phosphate inhibits TLR4-dependent RANTES production in macrophages. Int J Biochem Cell Biol.

[204] Grant BD, Donaldson JG (2009). Pathways and mechanisms of endocytic recycling. Nat Rev Mol Cell Biol.

[205] Naslavsky N, Caplan S (2018). The enigmatic endosome - sorting the ins and outs of endocytic trafficking. J Cell Sci.

[206] Green EG, Ramm E, Riley NM, Spiro DJ, Goldenring JR, Wessling-Resnick M (1997). Rab11 is associated with transferrin-containing recycling compartments in K562 cells. Biochem Biophys Res Commun.

[207] Yu B, Hailman E, Wright SD (1997). Lipopolysaccharide binding protein and soluble CD14 catalyze exchange of phospholipids. J Clin Invest.

[208] Fassbender K, Walter S, Kühl S, Landmann R, Ishii K, Bertsch T, Stalder AK, Muehlhauser F, Liu Y, Ulmer AJ, Rivest S, Lentschat A, Gulbins E, Jucker M, Staufenbiel M, Brechtel K, Walter J, Multhaup G, Penke B, Adachi Y, Hartmann T, Beyreuther K (2004). The LPS receptor (CD14) links innate immunity with Alzheimer’s disease. FASEB J.

[209] Zanoni I, Tan Y, Di Gioia M, Broggi A, Ruan J, Shi J, Donado CA, Shao F, Wu H, Springstead JR, Kagan JC (2016). An endogenous caspase-11 ligand elicits interleukin-1 release from living dendritic cells. Science.

[210] Prymas K, Świątkowska A, Traczyk G, Ziemlińska E, Dziewulska A, Ciesielska A, Kwiatkowska K (2020). Sphingomyelin synthase activity affects TRIF-dependent signaling of Toll-like receptor 4 in cells stimulated with lipopolysaccharide. Biochim Biophys Acta Mol Cell Biol Lipids.

[211] Gupta GD, Dey G, Swetha MG, Ramalingam B, Shameer K, Thottacherry JJ, Kalappurakkal JM, Howes MT, Chandran R, Das A, Menon S, Parton RG, Sowdhamini R, Thattai M, Mayor S (2014). Population distribution analyses reveal a hierarchy of molecular players underlying parallel endocytic pathways. PLoS ONE.

[212] van der Mark VA, Ghiboub M, Marsman C, Zhao J, van Dijk R, Hiralall JK, Ho-Mok KS, Castricum Z, de Jonge WJ, Oude Elferink RPJ, Paulusma CC (2017). Phospholipid flippases attenuate LPS-induced TLR4 signaling by mediating endocytic retrieval of Toll-like receptor 4. Cell Mol Life Sci.

[213] Lakshminarayan R, Wunder C, Becken U, Howes MT, Benzing C, Arumugam S, Sales S, Ariotti N, Chambon V, Lamaze C, Loew D, Shevchenko A, Gaus K, Parton RG, Johannes L (2014). Galectin-3 drives glycosphingolipid-dependent biogenesis of clathrin-independent carriers. Nat Cell Biol.

[214] Kalia M, Kumari S, Chadda R, Hill MM, Parton RG, Mayor S (2006). Arf6-independent GPI-anchored protein-enriched early endosomal compartments fuse with sorting endosomes via a Rab5/phosphatidylinositol-3′-kinase–dependent machinery. Mol Biol Cell.

[215] Guerville M, Boudry G (2016). Gastrointestinal and hepatic mechanisms limiting entry and dissemination of lipopolysaccharide into the systemic circulation. Am J Physiol Gastrointest Liver Physiol.

[216] Weinrauch Y, Katz SS, Munford RS, Elsbach P, Weiss J (1999). Deacylation of purified lipopolysaccharides by cellular and extracellular components of a sterile rabbit peritoneal inflammatory exudate. Infect Immun.

[217] Minasyan H (2019). Sepsis: mechanisms of bacterial injury to the patient. Scand J Trauma Resusc Emerg Med.

[218] Levels JH, Abraham PR, van den Ende A, van Deventer SJ (2001). Distribution and kinetics of lipoprotein-bound endotoxin. Infect Immun.

[219] Vreugdenhil ACE, Rousseau CH, Hartung T, Greve JWM (1950). van ’t Veer C, Buurman WA (2003) Lipopolysaccharide (LPS)-binding protein mediates LPS detoxification by chylomicrons. J Immunol.

[220] Bas S, Gauthier BR, Spenato U, Stingelin S, Gabay C (2004). CD14 Is an Acute-Phase Protein. J Immunol.

[221] Memar MY, Baghi HB (2019) Presepsin: a promising biomarker for the detection of bacterial infections. biomed pharmacother biomedecine pharmacother 111:649–656. 10.1016/j.biopha.2018.12.124 10.1016/j.biopha.2018.12.124 30611989

[222] Thompson PA, Tobias PS, Viriyakosol S, Kirkland TN, Kitchens RL (2003). Lipopolysaccharide (LPS)-binding protein inhibits responses to cell-bound LPS. J Biol Chem.

[223] Kitchens RL, Thompson PA, Viriyakosol S, O’Keefe GE, Munford RS (2001). Plasma CD14 decreases monocyte responses to LPS by transferring cell-bound LPS to plasma lipoproteins. J Clin Invest.

[224] Shao B, Munford RS, Kitchens R, Varley AW (2012). Hepatic uptake and deacylation of the LPS in bloodborne LPS-lipoprotein complexes. Innate Immun.

[225] Topchiy E, Cirstea M, Kong HJ, Boyd JH, Wang Y, Russell JA, Walley KR (2016). Lipopolysaccharide is cleared from the circulation by hepatocytes via the low density lipoprotein receptor. PLoS ONE.

[226] Suzuki K, Murakami T, Hu Z, Tamura H, Kuwahara-Arai K, Iba T (1950). Nagaoka I (2016) Human host defense cathelicidin peptide LL-37 enhances the lipopolysaccharide uptake by liver sinusoidal endothelial cells without cell activation. J Immunol.

[227] Yao Z, Mates JM, Cheplowitz AM, Hammer LP, Maiseyeu A, Phillips GS, Wewers MD, Rajaram MVS, Robinson JM, Anderson CL (1950). Ganesan LP (2016) Blood-borne lipopolysaccharide is rapidly eliminated by liver sinusoidal endothelial cells via high-density lipoprotein. J Immunol.

[228] Yokoyama S, Cai Y, Murata M, Tomita T, Yoneda M, Xu L, Pilon AL, Cachau RE, Kimura S (2018) A novel pathway of LPS uptake through syndecan-1 leading to pyroptotic cell death. eLife 7.10.7554/eLife.37854 10.7554/eLife.37854 PMC6286126 30526845

[229] Dunzendorfer S, Lee H-K, Soldau K, Tobias PS (2004). TLR4 is the signaling but not the lipopolysaccharide uptake receptor. J Immunol.

[230] Scott MJ, Billiar TR (2008). Beta2-integrin-induced p38 MAPK activation is a key mediator in the CD14/TLR4/MD2-dependent uptake of lipopolysaccharide by hepatocytes. J Biol Chem.

[231] Lu M (1950). Munford RS (2011) The transport and inactivation kinetics of bacterial lipopolysaccharide influence its immunological potency in vivo. J Immunol.

[232] Qian G, Jiang W, Zou B, Feng J, Cheng X, Gu J, Chu T, Niu C, He R, Chu Y, Lu M (2018). LPS inactivation by a host lipase allows lung epithelial cell sensitization for allergic asthma. J Exp Med.

[233] Zou B, Jiang W, Han H, Li J, Mao W, Tang Z, Yang Q, Qian G, Qian J, Zeng W, Gu J, Chu T, Zhu N, Zhang W, Yan D, He R, Chu Y, Lu M (2017). Acyloxyacyl hydrolase promotes the resolution of lipopolysaccharide-induced acute lung injury. PLoS Pathog.

[234] Gorelik A, Illes K, Nagar B (2018). Crystal structure of the mammalian lipopolysaccharide detoxifier. Proc Natl Acad Sci.

[235] Kaliannan K, Hamarneh SR, Economopoulos KP, Nasrin Alam S, Moaven O, Patel P, Malo NS, Ray M, Abtahi SM, Muhammad N, Raychowdhury A, Teshager A, Mohamed MMR, Moss AK, Ahmed R, Hakimian S, Narisawa S, Millán JL, Hohmann E, Warren HS, Bhan AK, Malo MS, Hodin RA (2013). Intestinal alkaline phosphatase prevents metabolic syndrome in mice. Proc Natl Acad Sci.

[236] Fink MP (2014). Animal models of sepsis. Virulence.

[237] Shao B, Lu M, Katz SC, Varley AW, Hardwick J, Rogers TE, Ojogun N, Rockey DC, Dematteo RP, Munford RS (2007). A host lipase detoxifies bacterial lipopolysaccharides in the liver and spleen. J Biol Chem.

[238] Parker H, Bigger BW (2019). The role of innate immunity in mucopolysaccharide diseases. J Neurochem.

[239] Simonaro CM (2016) Lysosomes, lysosomal storage diseases, and inflammation.J Inborn Errors Metab Screen 4:1–8. 10.1177/2326409816650465

[240] Simonaro CM, D’Angelo M, He X, Eliyahu E, Shtraizent N, Haskins ME, Schuchman EH (2008). Mechanism of glycosaminoglycan-mediated bone and joint disease: implications for the mucopolysaccharidoses and other connective tissue diseases. Am J Pathol.

[241] Simonaro CM, Ge Y, Eliyahu E, He X, Jepsen KJ, Schuchman EH (2010). Involvement of the Toll-like receptor 4 pathway and use of TNF-alpha antagonists for treatment of the mucopolysaccharidoses. Proc Natl Acad Sci.

[242] Ausseil J, Desmaris N, Bigou S, Attali R, Corbineau S, Vitry S, Parent M, Cheillan D, Fuller M, Maire I, Vanier M-T, Heard J-M (2008). Early neurodegeneration progresses independently of microglial activation by heparan sulfate in the brain of mucopolysaccharidosis IIIB mice. PLoS ONE.

[243] Suzuki M, Sugimoto Y, Ohsaki Y, Ueno M, Kato S, Kitamura Y, Hosokawa H, Davies JP, Ioannou YA, Vanier MT, Ohno K, Ninomiya H (2007). Endosomal accumulation of Toll-like receptor 4 causes constitutive secretion of cytokines and activation of signal transducers and activators of transcription in Niemann-Pick disease type C (NPC) fibroblasts: a potential basis for glial cell activation in the NPC brain. J Neurosci.

[244] Hauber H-P, Tulic MK, Tsicopoulos A, Wallaert B, Olivenstein R, Daigneault P, Hamid Q (2005). Toll-like receptors 4 and 2 expression in the bronchial mucosa of patients with cystic fibrosis. Can Respir J.

[245] Bruscia EM, Zhang P-X, Ferreira E, Caputo C, Emerson JW, Tuck D, Krause DS, Egan ME (2009). Macrophages directly contribute to the exaggerated inflammatory response in cystic fibrosis transmembrane conductance regulator−/− mice. Am J Respir Cell Mol Biol.

[246] Muir A, Soong G, Sokol S, Reddy B, Gomez MI, Van Heeckeren A, Prince A (2004). Toll-like receptors in normal and cystic fibrosis airway epithelial cells. Am J Respir Cell Mol Biol.

[247] Kelly C, Canning P, Buchanan PJ, Williams MT, Brown V, Gruenert DC, Elborn JS, Ennis M, Schock BC (2013). Toll-like receptor 4 is not targeted to the lysosome in cystic fibrosis airway epithelial cells. Am J Physiol Lung Cell Mol Physiol.

[248] Bruscia EM, Zhang P-X, Satoh A, Caputo C, Medzhitov R, Shenoy A, Egan ME (1950). Krause DS (2011) Abnormal trafficking and degradation of TLR4 underlie the elevated inflammatory response in cystic fibrosis. J Immunol.

[249] Seo SH, Hwang SM, Ko JM, Ko JS, Hyun YJ, Cho SI, Park H, Kim SY, Seong M-W, Park SS (2015). Identification of novel mutations in the VPS33B gene involved in arthrogryposis, renal dysfunction, and cholestasis syndrome. Clin Genet.

[250] Trotta T, Porro C, Calvello R, Panaro MA (2014). Biological role of Toll-like receptor-4 in the brain. J Neuroimmunol.

[251] Walter S, Letiembre M, Liu Y, Heine H, Penke B, Hao W, Bode B, Manietta N, Walter J, Schulz-Schüffer W, Fassbender K (2007). Role of the Toll-Like receptor 4 in neuroinflammation in Alzheimer’s disease. Cell Physiol Biochem.

[252] Reed-Geaghan EG, Savage JC, Hise AG, Landreth GE (2009). CD14 and Toll-like receptors 2 and 4 are required for fibrillar Aβ-stimulated microglial activation. J Neurosci.

[253] Frank S, Copanaki E, Burbach GJ, Müller UC, Deller T (2009). Differential regulation of Toll-like receptor mRNAs in amyloid plaque-associated brain tissue of aged APP23 transgenic mice. Neurosci Lett.

[254] Tang S-C, Lathia JD, Selvaraj PK, Jo D-G, Mughal MR, Cheng A, Siler DA, Markesbery WR, Arumugam TV, Mattson MP (2008). Toll-like receptor-4 mediates neuronal apoptosis induced by amyloid beta-peptide and the membrane lipid peroxidation product 4-hydroxynonenal. Exp Neurol.

[255] Casula M, Iyer AM, Spliet WGM, Anink JJ, Steentjes K, Sta M, Troost D, Aronica E (2011). Toll-like receptor signaling in amyotrophic lateral sclerosis spinal cord tissue. Neuroscience.

[256] Letiembre M, Liu Y, Walter S, Hao W, Pfander T, Wrede A, Schulz-Schaeffer W, Fassbender K (2009). Screening of innate immune receptors in neurodegenerative diseases: a similar pattern. Neurobiol Aging.

[257] Liu Y, Walter S, Stagi M, Cherny D, Letiembre M, Schulz-Schaeffer W, Heine H, Penke B, Neumann H, Fassbender K (2005). LPS receptor (CD14): a receptor for phagocytosis of Alzheimer’s amyloid peptide. Brain J Neurol.

[258] Martin E, Boucher C, Fontaine B, Delarasse C (2017). Distinct inflammatory phenotypes of microglia and monocyte-derived macrophages in Alzheimer’s disease models: effects of aging and amyloid pathology. Aging Cell.

[259] Reed-Geaghan EG, Reed QW, Cramer PE, Landreth GE (2010). Deletion of CD14 attenuates Alzheimer’s disease pathology by influencing the brain’s inflammatory milieu. J Neurosci.

[260] Wallings RL, Humble SW, Ward ME, Wade-Martins R (2019). Lysosomal dysfunction at the centre of Parkinson’s disease and frontotemporal dementia/amyotrophic lateral sclerosis. Trends Neurosci.

[261] Wang C, Telpoukhovskaia MA, Bahr BA, Chen X, Gan L (2018). Endo-lysosomal dysfunction: a converging mechanism in neurodegenerative diseases. Curr Opin Neurobiol.

[262] Schmid W, Novacek G, Vogelsang H, Papay P, Primas C, Eser A, Panzer S (2017). Platelets Toll-like receptor-4 in Crohns disease. Eur J Clin Invest.

[263] Roviezzo F, Sorrentino R, Terlizzi M, Riemma MA, Iacono VM, Rossi A, Spaziano G, Pinto A, D’Agostino B, Cirino G (2017). Toll-Like Receptor 4 is essential for the expression of sphingosine-1-phosphate-dependent asthma-like disease in mice. Front Immunol.

[264] Alibashe-Ahmed M, Brioudes E, Reith W, Bosco D, Berney T (2019). Toll-like receptor 4 inhibition prevents autoimmune diabetes in NOD mice. Sci Rep.

[265] Watanabe S, Kumazawa Y, Inoue J (2013). Liposomal lipopolysaccharide initiates TRIF-dependent signaling pathway independent of CD14. PLoS ONE.

